# Dihydroartemisinin restores the immunogenicity and enhances the anticancer immunosurveillance of cisplatin by activating the PERK/eIF2α pathway

**DOI:** 10.1186/s13578-024-01254-0

**Published:** 2024-08-01

**Authors:** Yumei Li, Pei Ma, Jingxia Li, Feng Wu, Mengfei Guo, E Zhou, Siwei Song, Sufei Wang, Shuai Zhang, Yang Jin

**Affiliations:** 1 Department of Respiratory and Critical Care Medicine, Hubei Province Clinical Research Center for Major Respiratory Diseases, Key Laboratory of Pulmonary Diseases of National Health Commission, Union Hospital, Tongji Medical College, Huazhong University of Science and Technology, Wuhan, 430022 Hubei China; 2 The Ministry of Education Key Laboratory of Biological Targeted Therapy, Tongji Medical College, Union Hospital, Huazhong University of Science and Technology, Wuhan, China; 3 Hubei Province Engineering Research Center for Tumor-Targeted Biochemotherapy, Union Hospital, Tongji Medical College, Huazhong University of Science and Technology, Wuhan, China; 4 State Key Laboratory of Respiratory Disease, The First Affiliated Hospital of Guangzhou Medical University, Guangzhou, China

**Keywords:** Immunosurveillance, Immunogenicity, Cisplatin, Dihydroartemisinin, Endoplasmic reticulum stress

## Abstract

**Background:**

Immunosurveillance is pivotal in the effectiveness of anticancer therapies and tumor control. The ineffectiveness of cisplatin in activating the immunosurveillance is attributed to its lack of adjuvanticity resulting from its inability to stimulate endoplasmic reticulum stress. Dihydroartemisinin demonstrates the anti-tumor effects through various mechanisms, including the activation of the endoplasmic reticulum stress. This study aimed to develop a novel strategy to enhance the immunogenicity of dying tumor cells by combining cisplatin with dihydroartemisinin, thereby triggering effective anti-tumor immunosurveillance and improving the efficacy of cisplatin in clinical practice.

**Methods:**

Lewis lung carcinoma (LLC) and CT26 colon cancer cell lines and subcutaneous tumor models were used in this study. The importance of immunosurveillance was validated in both immunocompetent and immunodeficient mouse models. The ability of dihydroartemisinin and cisplatin therapy to induce immunogenic cell death and tumor growth control in vivo was validated by prophylactic tumor vaccination and therapeutic tumor models. The underlying mechanism was elucidated through the pharmaceutical or genetic intervention of the PERK/eIF2α pathway in vitro and in vivo.

**Results:**

Dihydroartemisinin enhanced the generation of reactive oxygen species in cisplatin-treated LLC and CT26 cancer cells. The combination treatment of dihydroartemisinin with cisplatin promoted cell death and ensured an optimal release of damage-associated molecular patterns from dying cancer cells, promoting the phagocytosis of dendritic cells. In the tumor vaccination model, we confirmed that dihydroartemisinin plus cisplatin treatment induced immunogenic cell death. Utilizing immunocompetent and immunodeficient mouse models, we further demonstrated that the combination treatment suppressed the tumor growth of CT26 colon cancer and LLC lung cancer, leading to an improved prognosis through the restoration of cytotoxic T lymphocyte responses and reinstatement of anti-cancer immunosurveillance in vivo. Mechanistically, dihydroartemisinin restored the immunogenicity of cisplatin by activating the adjuvanticity of damage-associated molecular patterns, such as calreticulin exposure, through the PERK/eIF2α pathway. Additionally, the inhibition of eIF2α phosphorylation attenuated the anti-tumor efficiency of C + D in vivo.

**Conclusions:**

We highlighted that dihydroartemisinin acts as an immunogenic cell death rescuer for cisplatin, activating anticancer immunosurveillance in a PERK/eIF2α-dependent manner and offering a strategy to enhance the anti-tumor efficacy of cisplatin in clinical practice.

**Supplementary Information:**

The online version contains supplementary material available at 10.1186/s13578-024-01254-0.

## Background

The incidence and mortality rates of malignant tumors are rapidly increasing in globally [1]. When cancer cells become invisible or actively evade immune system surveillance, malignant tumors can develop and progress, rendering treatments ineffective or resistant [2]. Successful anti-tumor therapy requires both direct cytotoxicity to cancer cells and immunologic surveillance of host tissues [3]. Immunogenic cell death (ICD) is a form of immunogenic apoptosis associated with activating an adaptive immune response [4]. In clinical practice, the application of ICD inducers has shown a positive correlation with overall survival and progression-free survival in patients with various cancers, including breast, colorectal, head and neck, hepatocellular carcinoma, melanoma, prostate, and ovarian cancers [5–11]. Over the past decades, it has been demonstrated that the induction of ICD depends on the antigenicity of the dying tumors and the adjuvanticity of damage-associated molecular patterns (DAMPs). These include the extracellular exposure of calreticulin (CALR) and other endoplasmic reticulum (ER) proteins in the pre-apoptotic stage, the secretion of adenosine triphosphate (ATP) in the apoptotic phase, and the release of high mobility group box 1 (HMGB1) in the late apoptotic stage by dying cells [4, 12–14]. Notably, these parameters seem to be sufficient to accurately predict drug-induced ICD [15].

Cisplatin (CDDP), a first-line chemotherapy drug for anti-tumor treatment in clinical practice, fails to induce ICD due to its inability to activate adaptive immunity during cell death [16]. In some cases, the immunogenicity of CDDP can be restored through combinations with other interventions. For example, thapsigargin (Tha) and tunicamycin restore the immunogenicity of CDDP-induced cancer cell death through ER stress-induced CALR exposure [17]. When combined with CDDP, pyridoxine (the active form of vitamin B6) can induce ICD in non-small cell lung cancer (NSCLC) both in vitro and in vivo [18]. In a previous study, we demonstrated that when combined with CDDP, ischemia/reperfusion injury induces ICD in lung cancer cells [19]. Consequently, exploring additional ICD inducers to enhance immunosurveillance is essential as it may offer more strategies for clinical anti-tumor therapy.

Known for their use in antimalarial treatment worldwide due to their proven biosafety in patients [20], artemisinin and its derivatives are also under investigation for controlling infections, inflammation, cancer, and other diseases [21]. Dihydroartemisinin (DHA), an active metabolite of artemisinin, exhibits anticancer effects in various tumors, including glioma [22], lung cancer [23], colon cancer [24], breast cancer [25], ovarian cancer [26], and pancreatic cancer [27] via a multitude of mechanisms, including reactive oxygen species (ROS) generation, autophagy, and ER stress [28–30]. However, the impact of DHA on immunosurveillance has not yet been explored. Therefore, this study aimed to investigate the potential of DHA to activate anti-tumor immunosurveillance and enhance the efficacy of the commonly-used chemotherapeutic cisplatin.

In this study, we reported that the combination treatment of CDDP with DHA (C + D) could induce ICD in vivo by tumor vaccine inoculation model. In vitro, DHA enhanced the release of CDDP-induced DAMPs and promoted the phagocytosis of bone marrow-derived dendritic cells (BMDCs). Furthermore, C + D therapy significantly inhibited tumor growth, depending on the activation of immunosurveillance in the tumor microenvironment (TME). Mechanistically, C + D therapy stimulates PERK-dependent eIF2α phosphorylation in ER stress and triggers CALR exposure, further enhancing effective anticancer immunosurveillance. Additionally, the inhibition of eIF2α phosphorylation attenuated the anti-tumor efficiency of C + D in vivo. Our findings establish the mechanistic foundation for the potential clinical applications of CDDP and DHA combination treatment in anticancer therapy.

## Methods

### Animals, cell culture, and chemicals

Female BALB/c, male BALB/c-nu, and male C57 mice (6–8 weeks old) were purchased from Beijing Vital River Laboratory Animal Technology Co., Ltd (Beijing, China). All mice were housed in a specific pathogen-free environment at the Animal Center of Huazhong University of Science and Technology, China. All animal experiments were approved by the Animal Care Committee of Tongji Medical College, Hua Zhong University of Science and Technology ([2018] IACUC number: 3106) and conducted in accordance with the approved protocol, the ARRIVE guidelines [31] and the National Institutes of Health guide for the care and use of laboratory animals.

Lewis lung carcinoma (LLC) and murine colorectal carcinoma (CT26) cells were purchased from the China Center for Type Culture Collection (Wuhan, China). LLC cells were cultured in DMEM and CT26 cells in RPMI 1640 medium, which were supplemented with 10% fetal bovine serum (FBS), l-glutamine (2 mM), pyruvate (1 mM), penicillin (100 U/mL), and streptomycin (100 µg/mL).

Cisplatin, dihydroartemisinin, and integrated stress response inhibitor (ISRIB) were purchased from Selleck (Shanghai, China), while thapsigargin was obtained from Sigma-Aldrich (St. Louis, MO, USA). According to the manufacturer’s instructions, these chemicals were dissolved in dimethyl sulfoxide (DMSO) or N, N-dimethylformamide (DMF), respectively.

### Quantification of apoptosis and cell death

Apoptosis and cell death were assessed by annexin V-fluorescein isothiocyanate/7-amino actinomycin D (7-AAD) staining (BD Biosciences, San Jose, CA, USA). Briefly, LLC and CT26 cells were treated with control solvent, CDDP, DHA, or C + D at a concentration of 100/150 µM CDDP or/and 10 µM DHA for 24 h, and the cells were collected, washed, and resuspended in 1× binding buffer. Subsequently, the anti-annexin V antibody and 7-AAD were co-stained at 4 ℃ for 15 min in the dark. All samples were analyzed using a BD LSR Fortessa X-20 flow cytometer (Becton Dickinson, Franklin Lakes, NJ, USA).

### ROS generation

According to the manufacturer’s instructions, ROS levels were measured using a ROS Assay Kit (Beyotime Biotechnology, China). The LLC and CT26 cells, treated as described above, were harvested and stained with 10 µM 2’, 7’-dichlorofluorescein-diacetate (DCFH-DA) for 20 min at 37 ℃ in the dark. The stained cells were washed with phosphate-buffered saline and analyzed using BD LSR Fortessa X-20 flow cytometry (Becton Dickinson, Franklin Lakes, NJ, USA).

### Analysis of CALR exposure

LLC and CT26 cells, cultured in the presence of the control solvent, CDDP, DHA, or C + D for 6 h, underwent analysis of extracellular CALR expression by flow cytometry as previously described [32]. Briefly, cells were fixed and incubated with an anti-CALR antibody (Abcam, ab2907), followed by incubation with a secondary antibody (Abcam, ab150077) for thirty minutes. 7-AAD was added to each sample after washing and incubating at 4 ℃ in the dark. The stained samples were analyzed by flow cytometry. Sample incubated without the primary anti-CALR antibody was used as isotype control, and the percentage of CALR exposure on the live (7-AAD stained negative) cells was determined.

### ATP assays

LLC and CT26 cells were cultured with four different treatments for 24 h. Cell supernatants were collected and centrifuged at 400 × g for 5 min followed by a second centrifugation at 2,000 × g for 10 min at 4 ℃. The supernatants were collected and deproteinized using perchloric acid and potassium hydroxide, and ATP levels were measured using an ATP colorimetric/fluorometric assay kit (Abcam, ab83355) according to the manufacturer’s instructions. Fluorescence was assessed using a FLUOstar OPTIMA multilabel reader (BMG LabTech, Germany).

### Detection of HMGB1 release

After treatment of LLC and CT26 cells for 24 h, cell supernatants were collected and centrifuged as described above. HMGB1 levels in the cell supernatants were quantified using an HMGB1 ELISA kit (Shino Test Corporation, Japan) following the manufacturer’s instructions and detected on a FLUOstar OPTIMA multilabel reader (BMG LabTech, Germany).

### Generation of bone marrow-derived dendritic cells

BMDCs were differentiated from bone marrow precursors according to previously published protocols [33]. Briefly, 2 × 10^6^ bone marrow cells were cultured in the complete RPMI 1640 medium, supplemented with 10% heat-inactivated FBS and mGM-CSF (20 ng/mL, Pepro Tech, NJ, USA). On the third day, a fresh complete culture medium was added. On the sixth and eighth days, half of the cultural supernatant was refreshed with the complete medium. On the ninth day, the BMDCs in the culture supernatant were harvested for further analysis.

### Phagocytosis assay

CT26 and LLC cells were labeled with 1 µM 1,1’-dioctadecyl-3,3,3’,3’-tetramethylindocarbocyanine perchlorate (DiL, Beyotime Biotechnology, China). After 20 h of treatment with control, DHA, CDDP, or C + D, the tumor cells were refreshed in a complete culture medium without drugs for 4 h. Subsequently, the cells were collected, washed, and co-cultured with 3,3’-dioctadecyloxacarbocyanine perchlorate (DiO, Beyotime Biotechnology, China)-labeled BMDCs or CD11c-labeled BMDCs for 14 h in a 1:2 ratio. The stained cells were visualized with the confocal laser scanning microscope (CLSM, Nikon-si-A1, Japan) or analyzed by flow cytometry.

### Western blot immunoassay

Protein was extracted by radioimmunoprecipitation assay buffer supplemented with phosphatase and protease inhibitors (Thermo fisher Scientific, Waltham, MA, USA). The protein was denatured at 100 ℃ for 10 min after adding a “protein Loading buffer” (Boster Biological Technology, China). Proteins were separated by sodium dodecyl sulfate-polyacrylamide gel electrophoresis and transferred to the polyvinylidene difluoride membrane. After being blocked with 5% skim milk powder in tris-buffered saline with 0.1% tween 20 (TBST), membranes were incubated with the following primary antibodies overnight at 4 ℃: anti-p-eIF2α (Cell Signaling Technology, 3398, 1:1,000), anti-eIF2α antibody (Cell Signaling Technology, 5324, 1:1,000), and anti-PERK antibody (Cell Signaling Technology, 3194, 1:1,000). Membranes were washed with TBST and HRP-conjugated secondary antibodies were incubated at room temperature for 1 h. After 3 washes with TBST, blots were detected using electrochemiluminescence (Thermo fisher Scientific, Waltham, MA, USA). β-actin was used as an internal reference.

### siRNA knockdown of EIF2ΑK3

According to the manufacturer’s instructions, 10 nM siGENOME mouse EIF2ΑK3 siRNA (Horizon, M-044901-01-0005) or siGENOME scrambled siRNA (Horizon, D-001206-13-05) was mixed with Lipofectamine™ RNAiMAX transfection reagent (Thermo fisher Scientific, Waltham, MA, USA) and diluted with Opti-MEM (Gibco, Grand Island, NY, USA). After treatment with non-coding or coding siRNA for 48 h, LLC and CT26 cells were seeded in appropriate plate with fresh culture medium for another 24 h, following different drug treatment.

### Vaccination assay of dying CT26 cells in vivo

CT26 cells were cultured with control solvents or chemotherapeutic drugs for 20 h, followed by 4 h of fresh complete culture medium without chemotherapeutic drugs to eliminate the residual drugs. For inhibiting the phosphorylation of eIF2α, CT26 cells were transfected with EIF2AK3 siRNA or scramble siRNA for 48 h before chemotherapeutic drug administration. Next, BALB/c-nu or BALB/c mice were randomized into three groups and subcutaneously inoculated with 3 × 10^6^ CT26 single tumor cell vaccines in 100 µl on the left flank, and an equal volume PBS without tumor cells was used as vaccine-negative control. All the tumor cell vaccines used in our vaccination model did not form primary tumors. One week later, untreated 5 × 10^5^ live CT26 cells in 100 µl were injected into the right flank. Tumor incidence and growth was observed for at least 30 days. Tumor-bearing mice were then euthanized and the tumors were stored for immunohistochemical staining after fixing, embedding, and sectioning.

### Histology and immunohistochemical (IHC) staining

Hematoxylin and eosin (H&E) staining was performed using standard procedures, and images were captured by light microscopy. Immunohistochemistry was conducted and analyzed as previously described [34]. Briefly, tumor tissue sections were dewaxed with xylene and rehydrated using different concentrations of ethanol. Antigen retrieval was achieved through a 20-minute microwave pretreatment in a sodium citrate solution, followed by cooling at room temperature. Endogenous peroxidase activity was blocked using 3% H_2_O_2_. After a 30-minute block using 5% BSA buffer, slides were incubated with primary anti-CD8 antibody (Abcam, ab209775, 1:50) or anti-Ki67 antibody (Servicebio, GB121141, 1:600), horseradish peroxidase-labeled secondary antibody, and diaminobenzidine substrate at the optimal concentration for IHC staining using an immunohistochemistry kit (Sangon Biotech, China), following the manufacturer’s instructions. Positive cells were counted in five fields on each slide using ImageJ software (http://imagej.nih.gov/ij/).

### Anti-tumor efficacy in mice bearing established tumors

Mice were anesthetized with 1% pentobarbital sodium, and a total of 5 × 10^5^ CT26 cells resuspended in 100 µl were subcutaneously inoculated into the right lower flank of BALB/c or BALB/c-nu mice. C57 mice were subcutaneously injected with 5 × 10^5^ LLC cells to generate flank-localized tumors. On the eighth day after-tumor injection, mice were randomized into four groups and intraperitoneally dosed with the solvent or chemotherapeutic drugs (3 mg/kg CDDP, 40 mg/kg DHA) once every other day for a total of four doses. For the eIF2α phosphorylation inhibition, vector or 2.5 mg/Kg ISRIB dissolved in corn oil was intraperitoneal (i.p.) injected once a day for two days before the C + D treatment, and then with ISRIB alone or the chemotherapy combination every other day from the eighth day. Body weight was monitored during treatment; tumor growth was measured with a digital caliper; and tumor diameter was calculated using the formula: V = 1/2a × b^2^ (where ‘a’ is the tumor length and ‘b’ is the tumor width). On the 18th day, tumor-bearing BALB/c mice were euthanized, and tumor tissue was stripped, weighed, and stored for TME analysis. After fixing, embedding, and sectioning the tumors, tumor slices were used for H&E, IHC, and terminal deoxynucleotidyl transferase-mediated dUTP nick-end labeling (TUNEL) staining. For survival analysis, mice were considered at the endpoint when tumor volume reached 2000 mm^3^.

### TUNEL assay

Tumor tissue slices were treated using an in situ cell death detection kit (Roche, Switzerland) according to the manufacturer’s instructions. In this experiment, apoptotic cells were stained green and analyzed through CLSM (Nikon-si-A1, Japan).

### Flow cytometry analysis for TME

To assess the proportion of immunocytes in the TME, flow cytometry analysis was performed as previously described [19]. Tumors were dissected and incubated in a buffer containing 2% FBS, DNAse I (0.02 mg/mL), and collagenase IV (2 mg/mL) (all from Sigma-Aldrich, St. Louis, MO, USA) for 1 h at 37 °C. Spleens and inguinal draining lymph nodes were homogenized. Red blood cells were lysed and the single-cell suspension was obtained through a 70 μm cell strainer. For surface staining, single cells were blocked with anti-CD16/CD32 antibodies and stained with the surface antibodies for 30 min at 4 ℃. Dead cells were excluded from all stained samples using the Zombie Aqua Fixable Viability Cell Staining Kit.

For intracellular detection of interferon-gamma (IFN-γ) in tumor-infiltrating lymphocytes (TILs), cells were cultured with phorbol myristate acetate (50 ng/mL, Sigma Aldrich), ionomycin (1 µg/mL, Sigma-Aldrich), and brefeldin A (1 µl/mL, BioLegend, San Diego, CA, USA) for 5 h at 37 °C. For intracellular staining, surface-labeled cells were fixed and permeabilized with Foxp3/Transcription Factor Staining Buffer Set (eBioscience, San Diego, CA, USA) and stained with intracellular antibodies. The stained cells were acquired using a BD LSR Fortessa X-20 flow cytometer (Becton Dickinson, Franklin Lakes, NJ, USA). The data were analyzed using FlowJo_V10 software. All antibodies were purchased from BD Pharmingen, eBioscience, or BioLegend; the details of these antibodies are shown in Table S1.

### Statistical analysis

All statistical analyses were performed using GraphPad Prism software (v.8.0, San Diego, CA, USA). All statistics were two-tailed. The Shapiro–Wilk test was used to test whether the data followed a normal distribution, and Levene’s F test was used to test whether the data exhibited variance homogeneity. The data that do not follow the normal distribution were log-transformed to base 10 logarithms (log10) to approach a normal distribution.

## Results

### C + D enhances ROS generation and apoptosis of tumor cells

**Fig. 1:**
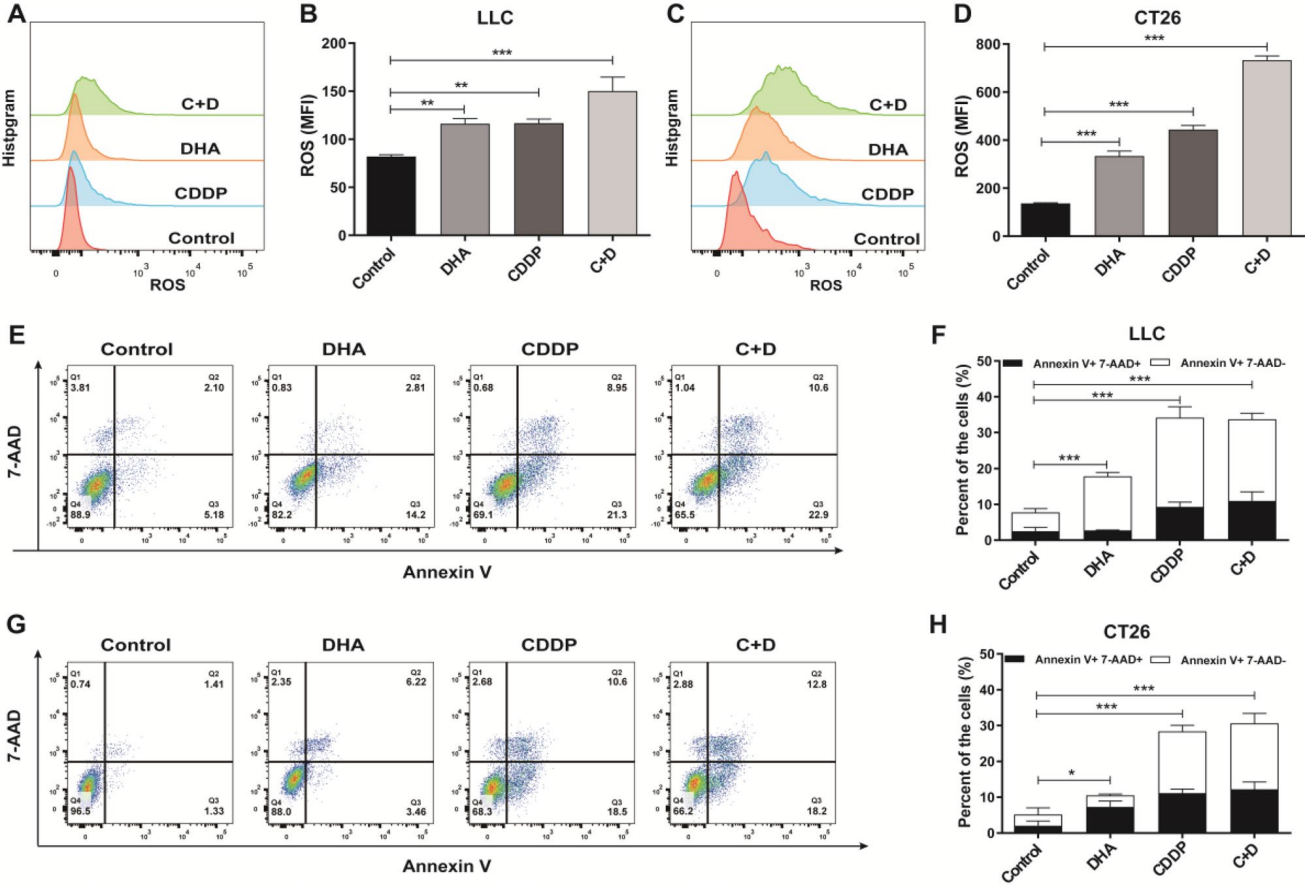
ROS generation and apoptosis of tumor cells induced by C + D treatment. LLC and CT26 cells were treated with control solvent, CDDP (100/150 µM), DHA (10 µM), and C + D for 24 h respectively, and were collected for further staining. (**A**–**D**) ROS generation in these cells was stained with 2’, 7’-dichlorofluorescein-diacetate (DCFH-DA) and analyzed by flow cytometry. **A** and **C** depict representative histograms of LLC and CT26 cells respectively, whereas **B** and **D** show quantitative data. (**E–H**) The apoptosis and cell death rates of treated tumor cells were co-stained with 7-AAD and Annexin V and analyzed by flow cytometry. Representative dot plots and statistically quantified data of (**E, F**) LLC cells and (**G, H**) CT26 cells are shown. White and black columns illustrate the percentage of dying and dead cells. Data are shown as mean ± SEM (*n* = 3), and statistical analyses were performed with a two-tailed unpaired Student’s t-test. *P value < 0.05, **P value < 0.01, ***P value < 0.001

We firstly validated cell death by assessing the ROS generation and apoptosis of LLC and CT26 cells. The data showed that compared with the control group, DHA or cisplatin individually promoted ROS generation in tumor cells and further enhanced ROS generation when combined in LLC and CT26 tumor cells (Fig. 1A–D). The cisplatin alone or C + D treatment significantly increased the percentage of apoptosis/necrosis of LLC and CT26 tumor cells, while DHA alone slightly increased apoptosis and cell death compared to the control group (Fig. 1E–H). Altogether, these results established that cisplatin and DHA combination treatment promoted cancer cell death.

### Identification of C + D as a candidate ICD inducer

**Fig. 2:**
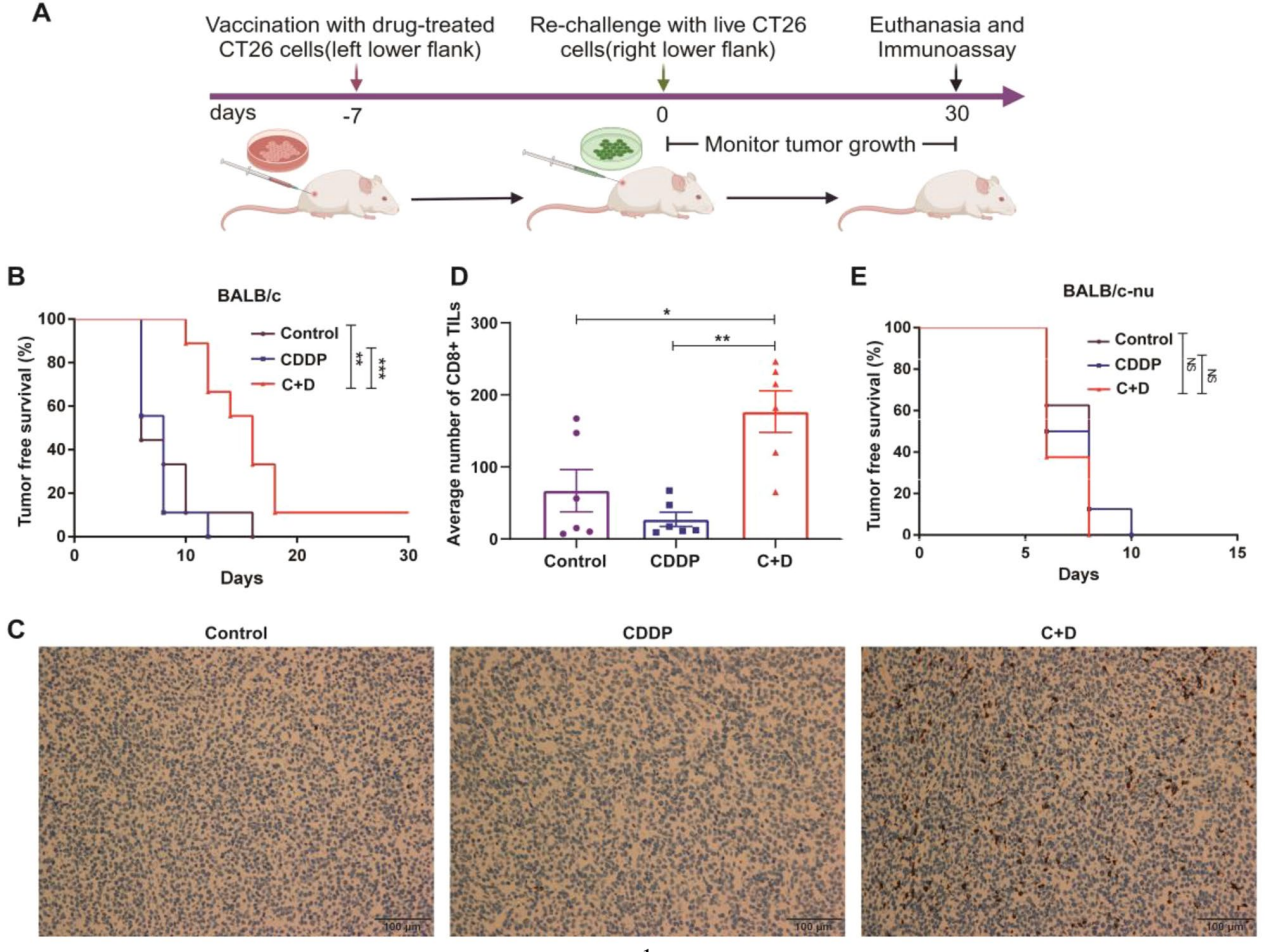
Identification of C + D as a candidate ICD inducer. (**A**) Experimental design for the prophylactic CT26 tumor cells vaccination model. CT26 cells treated with CDDP or C + D for 20 h and refreshed for 4 h with the complete medium were vaccinated into the BALB/c or BALB/c-nu mice, which were subcutaneously rechallenged with live CT26 cells after 7 days. PBS without tumor cells was applied as the vaccine-negative control. The tumor initiation and progression were monitored for 30 days. (**B**) Tumor-free survival of BALB/c mice after being injected with CDDP and C + D treated CT26 tumor vaccine (*n* = 9). (**C, D**) Endpoint tumors from vaccine-treated BALB/c mice were stained for anti-CD8 antibody. (**C**)The representative IHC staining images of CD8^+^ TILs in tumor tissues, as indicated by cells in brown color (scale bar, 100 μm). (**D**) Bar graphs represent the average number of CD8^+^ TILs from the tumor tissues. (**E**) The tumor-free survival of BALB/c-nu mice after being injected with CDDP, and C + D treated CT26 tumor vaccine (*n* = 8). The data are shown as mean ± SEM (*n* = 6), and statistical analyses were performed with one-way ANOVA followed by Holm–Sidak’s multiple comparisons test. For Kaplan–Meier, *P* values were determined by the log-rank test. *P value < 0.05, **P value < 0.01, ***P value < 0.001, NS *P* value > 0.05

We investigated whether C + D treatment effectively induced ICD in a syngeneic animal vaccination model (Fig. 2A). According to the guiding principle for reducing the use of experimental animals in the “Guide to the Care and Use of Experimental Animals”, the CT26 tumor model was only selected because the immunogenicity of CT26 cells was stronger than that of LLC cells. In addition, single DHA treatment was excluded from survival studies because the DHA-treated CT26 vaccine formed primary tumors in mice (data not shown).

The result showed that the C + D-treated CT26 vaccines delayed tumor initiation and prolonged tumor-free survival in immunocompetent BALB/c mice compared to the control group and CDDP alone (*n* = 6, Fig. 2B). Moreover, CD8^+^ TILs in endpoint tumors from vaccinated immunocompetent mice were detected using IHC staining. The results of CD8 staining showed that CDDP alone reduced the infiltration of CD8^+^ TILs, whereas the C + D treatment augmented the recruitment and infiltration of CD8^+^ TILs compared with the control group (Fig. 2C, D). In contrast, the protective effect of the C + D-treated CT26 vaccine was not observed in immunocompromised BALB/c-nu mice (*n* = 6, Fig. 2E). Thus, C + D therapy induced the ICD, promoted anti-tumor immunosurveillance, delayed tumor initiation, and prolonged tumor-free survival in immunocompetent mice.

### DHA induces DAMPs release and BMDCs phagocytosis

**Fig. 3:**
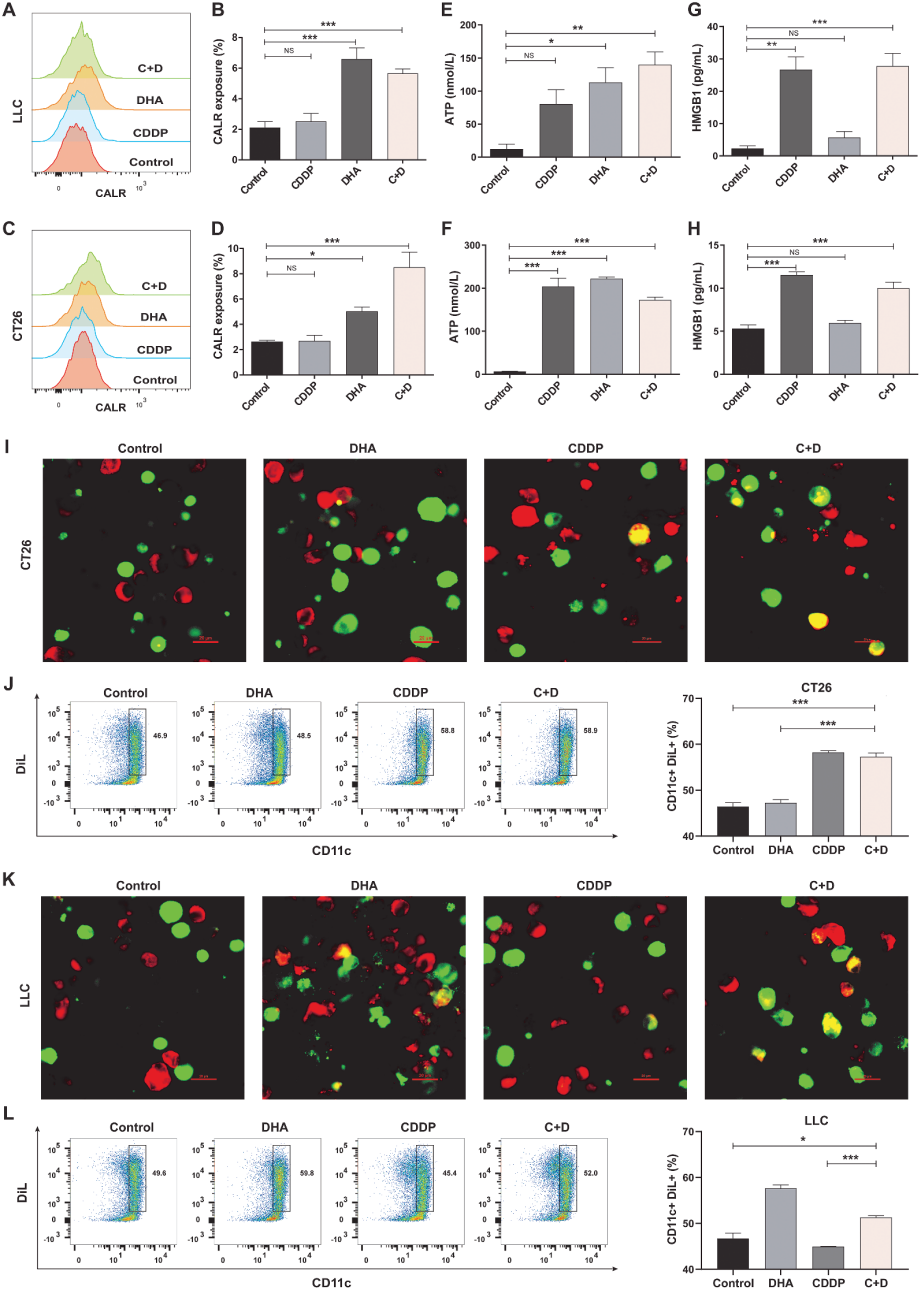
Damage-associated molecular patterns (DAMPs) released by C + D treated tumor cells and BMDCs phagocytosis. (**A–D**) The extracellular expression of CALR on the tumor cells after treatment with control solvent, CDDP, DHA, and C + D for 6 h was followed by CALR staining and analysis by flow cytometry. Representative histograms and statistically quantified data for (**A, B**) LLC cells and (**C, D**) CT26 cells are shown. (**E–G**) The tumor cells were treated with the above drugs for 24 h, and the supernatant was collected for further analysis. The extracellular ATP released by (**E**) LLC cells and (**F**) CT26 cells were determined by a luciferase-based assay. The release of HMGB1 (**G**) LLC and (**H**) CT26 cells was evaluated using an HMGB1-specific ELISA kit. (**I–L**) Uptake of drug-treated tumor cells by BMDCs. The CT26 and LLC tumor cells were labeled with the dye DiL (red). The uptake of different drug-treated (**I**) CT26 cells and (**K**) LLC cells by DiO-labeled BMDCs (green) was identified by representative images under CLSM (scale bar, 20 μm). The uptake efficiency of drug-treated (**J**) CT26 cells and (**L**) LLC cells was further evaluated after co-cultured with FITC-labeled BMDCs for 14 h by flow cytometry; the left and right panels represent the representative dot plots and the statistically quantified data, respectively. These data are shown as mean ± SEM (*n* = 3), and statistical analyses were performed with one-way ANOVA followed by Holm–Sidak’s multiple comparisons test. For (**J, L**) the uptake efficiency of BMDCs, *P* values were assessed based on unpaired Student’s t-test. * *P* value < 0.05, *** *P* value < 0.001

Next, we evaluated the release of DAMPs after different treatments. Our data showed that compared with the control group, treatment with DHA alone or combined with CDDP significantly increased the CALR exposure on the surface of LLC and CT26 cells (Fig. 3A–D). Moreover, ATP secretion was increased in DHA alone or C + D treated-tumor cells, compared with untreated cells (Fig. 3E, F). HMGB1 levels in dying tumor cells indicated that CDDP alone or the C + D treatment caused elevated levels of HMGB1 in tumor cells (Fig. 3G, H).

To further assess the immunogenic properties of C + D-treated tumor cells, we tested the phagocytosis ability of BMDCs by coculturing with the treated tumor cells in vitro. DiL-labeled tumor cells (red) were cocultured with DiO-labeled BMDCs (green) or CD11c-labeled BMDCs at a ratio of 1:2 for 14 h for CLSM or flow cytometry. Compared with CT26 cells without drug treatment, C + D-treated CT26 cells were effectively engulfed by the BMDCs (Fig. 3I, J, and Figure S1A). Moreover, C + D treated LLC cells were more effectively engulfed by the BMDCs than live cells without drug intervention (Fig. 3K, L, and Figure S1B).

### Anti-tumor effect of C + D treatment in vivo relies on immunosurveillance

**Fig. 4:**
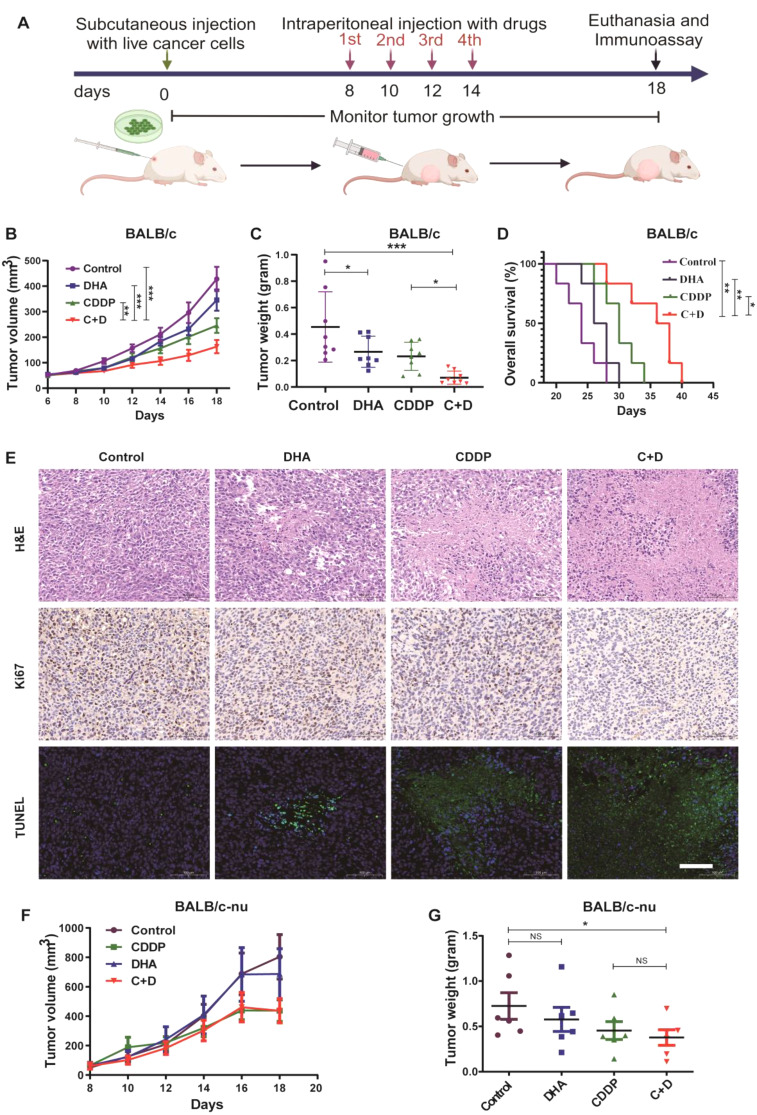
The anti-tumor effect of C + D treatment in vivo relies on immunosurveillance (**A**) Experimental design for the therapeutic subcutaneous tumor model. On the 8th day after tumor injection, the mice were randomized into four groups and intraperitoneally dosed with the solvent, 3 mg/kg CDDP, 40 mg/kg DHA alone, or a combination once every other day for four times. Body weight and tumor growth were monitored. (**B**) Tumor growth curves of BALB/c mice were monitored every other day after the drug intervention (*n* = 8). (**C**) Weight of the excised tumors from BALB/c mice after subcutaneous tumors were obtained from euthanized mice on the 18th day (*n* = 8). (**D**) The Kaplan–Meier survival curves of CT26 tumor-bearing BALB/c mice after the different treatments (*n* = 6). (**E**) Representative H&E, IHC staining for Ki67, and TUNEL staining (green, apoptotic cell; blue, DAPI) of excised tumor tissues from BALB/c mice after different treatments (scale bar, 200 μm). (**F**) The subcutaneous tumor volume of BALB/c-nu mice during the various treatments (*n* = 6). (**G**) Weight of the excised tumors of BALB/c-nu mice after subcutaneous tumors were obtained from euthanized mice on the 18th day (*n* = 6). The data are shown as mean ± SEM and statistical analyses were performed with one-way ANOVA followed by Holm–Sidak’s multiple comparisons test. For tumor growth curves, *P* values were calculated by two-way ANOVA followed by Holm–Sidak’s multiple comparisons test. For Kaplan–Meier, *P* values were determined by the log-rank test. *P value < 0.05, **P value < 0.01, ***P value < 0.001

The anti-tumor effect of C + D treatment was validated using a therapeutic mouse subcutaneous tumor model (Fig. 4A). In the BALB/c mouse model, our data showed that DHA alone partially reduced tumor growth compared to the control, and C + D therapy achieved the most significant tumor growth inhibition in comparison to the control and CDDP alone treated groups (*n* = 8; Fig. 4B, C and Figure S2A). Additionally, the body weight of BALB/c mice in the four groups showed no significant change, indicating the negligible additional systemic toxicity of the combination treatment (Figure S2B). To ensure our observed anti-tumor phenotype was not unique to the CT26 colon cancer cell line, we repeated our study paradigm in C57 mice with the LLC lung cancer cell line and treated them as previously outlined. As with the CT26 tumor model, a higher tumor inhibition was observed after C + D treatment in LLC tumor-bearing mice (*n* = 8; Figure S3A).

We also investigated the prognostic implications of C + D treatment. The contribution of C + D treatment to mouse survival was assessed by monitoring survival following two different tumor model cohorts of control, CDDP, DHA, or C + D treatment: one with the administration of CT26 cells in BALB/c mice for the tumor-bearing BALB/c model (*n* = 6) and a second trial following injection of LLC cells into C57 mice to establish the tumor-bearing C57 model (*n* = 8). BALB/c mice treated with C + D survived significantly longer compared to the other three groups (Fig. 4D). The trend of prolonged survival following C + D treatment was reproducible in a second and independent trial that used the LLC tumor-bearing C57 model (Figure S3B).

The anti-tumor effects of different treatments in BALB/c mice were further evaluated using H&E staining, the Ki67 assay, and the TUNEL assay. As illustrated in Fig. 4E, compared with the other groups, the tumor tissues treated with the C + D therapy exhibited massive necrosis (H&E staining) and presented the lowest proliferation (Ki67 staining) and highest apoptosis levels (TUNEL staining).

To further explore whether the anti-tumor effect of C + D therapy depends on anti-tumor immunosurveillance, we observed the tumor inhibition effect of C + D treatment on immunocompromised BALB/c-nu mice bearing established tumors, following the protocol shown in Fig. 4A. As expected, the tumor growth curves showed no significant difference in tumor volume of BALB/c-nu mice among four different treatments (Fig. 4F). However, DHA alone failed to inhibit tumor growth, and the privileged tumor inhibition effect of C + D treatment disappeared (*n* = 6; Fig. 4F, G and Figure S4). Collectively, these results highlighted that the success of C + D treatment depends on an intact immune status, implicating the importance of immune surveillance in tumor growth inhibition induced by C + D treatment.

### C + D treatment promotes anti-tumor immunosurveillance

**Fig. 5:**
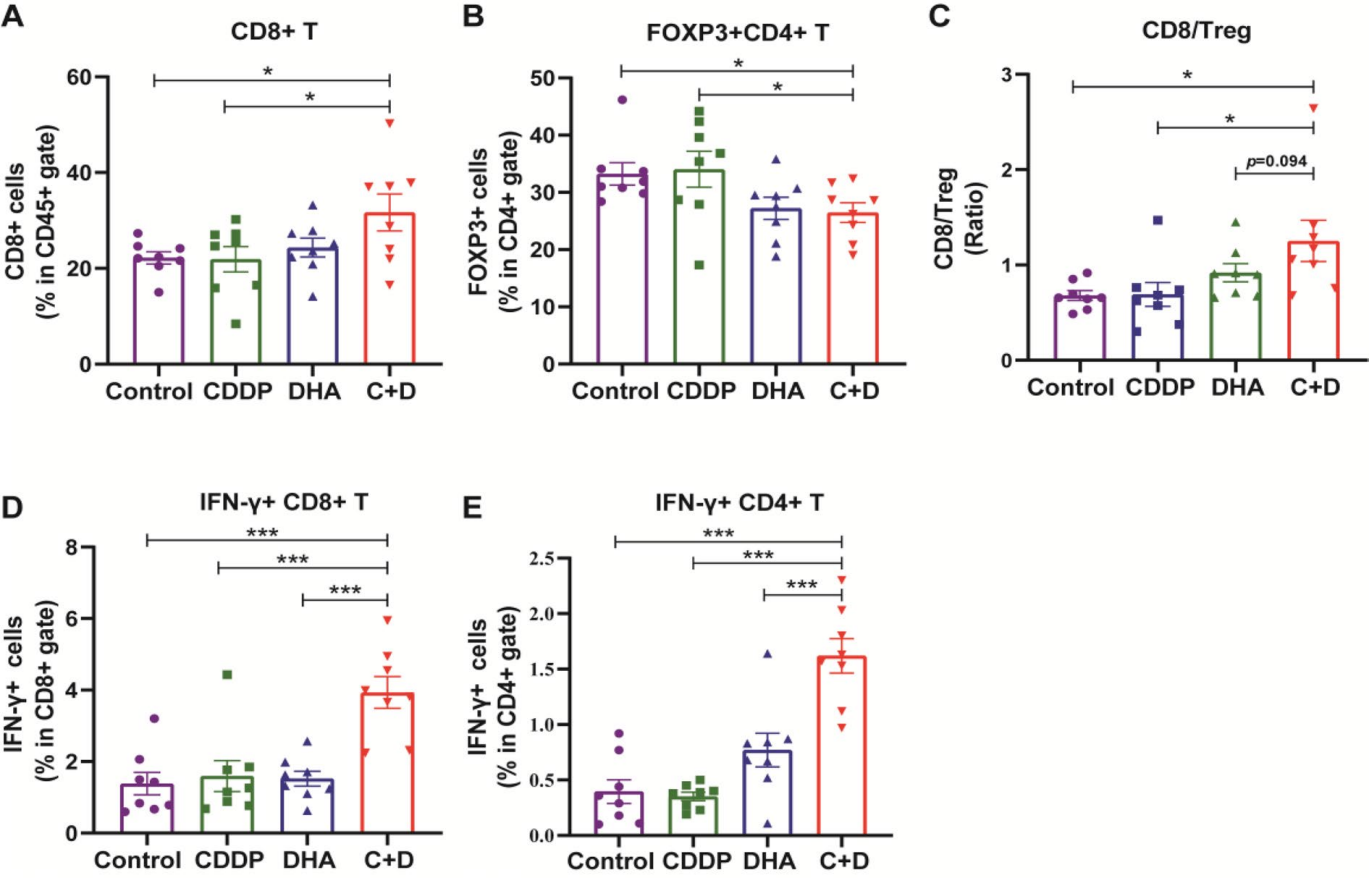
C + D treatment activates the anti-tumor immunity. Endpoint tumors from BALB/c mice on the 18th day after different treatments were collected for tumor-infiltrating lymphocyte analysis. Flow cytometry quantification of the (**A**) CD8^+^ T cells, (**B**) Tregs, (**C**) the ratio of CD8^+^ T/Treg, (**D**) IFNγ^+^CD8^+^ T cells, and (**E**) IFNγ^+^CD4^+^ T cells in the TME. These data are shown as mean ± SEM (*n* = 8), and statistical analyses were performed with one-way ANOVA followed by Holm–Sidak’s multiple comparisons test. *P value < 0.05, **P value < 0.01, ***P value < 0.001, NS *P* value > 0.05

To further investigate the anti-tumor immunity elicited by the C + D therapy in the TME, the proportion of TILs was examined by flow cytometry analysis (Figure S5). Our results showed that C + D treatment led to a higher proportion of CD8^+^ TILs while decreasing the infiltration of regulatory T cells (Tregs) compared with that of the control or CDDP treatment alone (Fig. 5A, B). Notably, the ratio of CD8^+^ T/Treg was the highest in the C + D treatment group compared with that in the other three groups (Fig. 5C). Meanwhile, compared with the control, DHA, or CDDP alone treatments, the levels of effector molecules IFN-𝛾 of CD4^+^ T and CD8^+^ T cells within the TME also dramatically increased in the C + D therapy group (Fig. 5D, E), implying that C + D treatment enhances the activation of effective T cells in vivo.

To provide a clear illustration of the immune reaction in the draining lymph node (DLN) and spleen, we utilized flow cytometry to quantify the macrophages and DCs. As shown in Figure S6, the M1 macrophages in DLN were significantly increased in the C + D therapy group compared with those in the control, DHA, and CDDP groups while there was no significant change in M2 macrophages. Compared with control or a single treatment, C + D treatment also showed upregulated co-stimulatory ligands CD86, MHCII, and CD86^+^MHCII^+^ expression on DCs, implying DCs maturation in the spleen (Figure S7). Collectively, these results clearly illustrated the activation of the immune landscape in TME, DLN, and the spleen, suggesting that the tumor growth-reducing effect of C + D therapy required the activation of immunosurveillance.

### C + D-induced CALR exposure relies on eIF2α phosphorylation of tumor cells

**Fig. 6:**
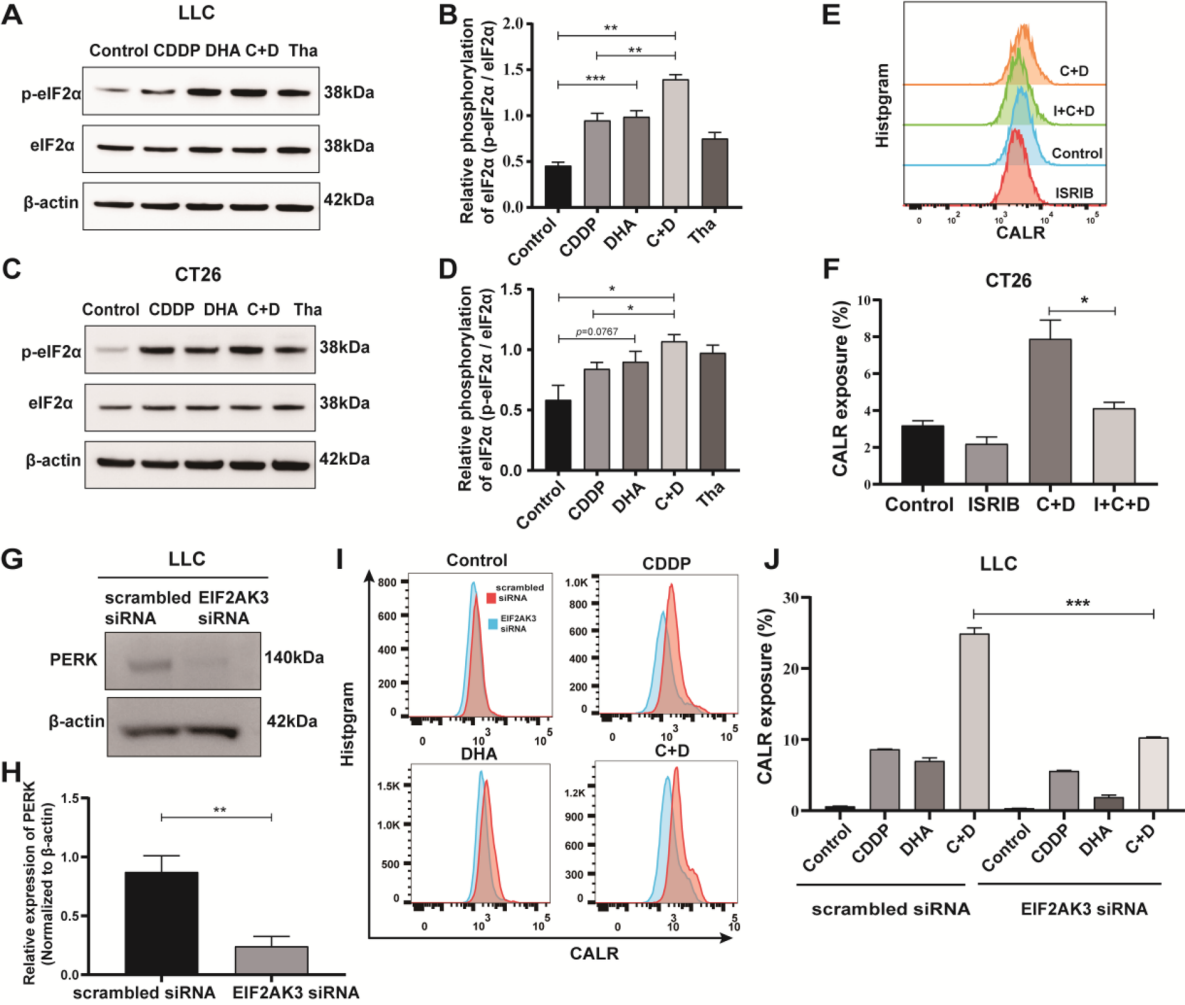
C + D-induced CALR exposure relies on eIF2α phosphorylation in tumor cells. (**A–D**) Phosphorylation status of eIF2α and expression level of eIF2α were determined after control solvent, CDDP, DHA, and C + D treatment of (**A, B**) LLC cells and (**C, D**) CT26 cells, and 250 nM Tha were selected as the positive control. Representative images are shown in A and C whereas B and D show quantitative data. (**E, F**) The CALR expression of CT26 cells was determined by western blot after C + D treatment with or without the 250 nM ISRIB pre-intervention for 30 min. (**G, H**) The expression of PERK was determined by Western blot after EIF2ΑK3 knockdown of LLC cells. (**I, J**) The CALR expression of EIF2ΑK3 knockdown of LLC cells after being treated with control, CDDP, DHA, or C + D at the indicated dose. Representative histograms are shown in **E** and **I**, whereas **F** and **J** show quantitative data. These data are shown as mean ± SEM (*n* = 3), and statistical analyses (**B, D**) were performed with one-way ANOVA followed by Holm–Sidak’s multiple comparisons test. For (**G, H**) the expression of PERK and (**F, J**) the expression of CALR, *P* values were assessed based on an unpaired Student’s t-test. * *P* value < 0.05, ** *P* value < 0.01, *** *P* value < 0.001, NS *P* value > 0.05

We investigated whether C + D-induced CALR exposure relies on eIF2α phosphorylation. Compared with the control treatment, DHA alone promoted eIF2α phosphorylation (Fig. 6A–D and Figure S8A, B). The C + D treatment further effectively triggered eIF2α phosphorylation at serine 51 compared to the control and CDDP alone (Fig. 6B, D). To determine if eIF2α phosphorylation plays a critical functional role in CALR exposure, we inhibited eIF2α phosphorylation by pharmaceutical or genetic methods to evaluate the extracellular expression of CALR. An integrated stress response inhibitor (ISRIB) was reported to reverse the effects of eIF2α phosphorylation [35]. We found that the presence of ISRIB inhibited the C + D-induced CALR exposure (Fig. 6E, F). Moreover, the genetic knockdown of EIF2ΑK3 siRNA in LLC cells reduced C + D-induced CALR exposure (Fig. 6G–J and Figure S8C) compared with scrambled siRNA. Altogether, C + D-induced CALR exposure relies on eIF2α phosphorylation.

### C + D treatment suppresses the tumor in a PERK/eIF2α-dependent manner

**Fig. 7:**
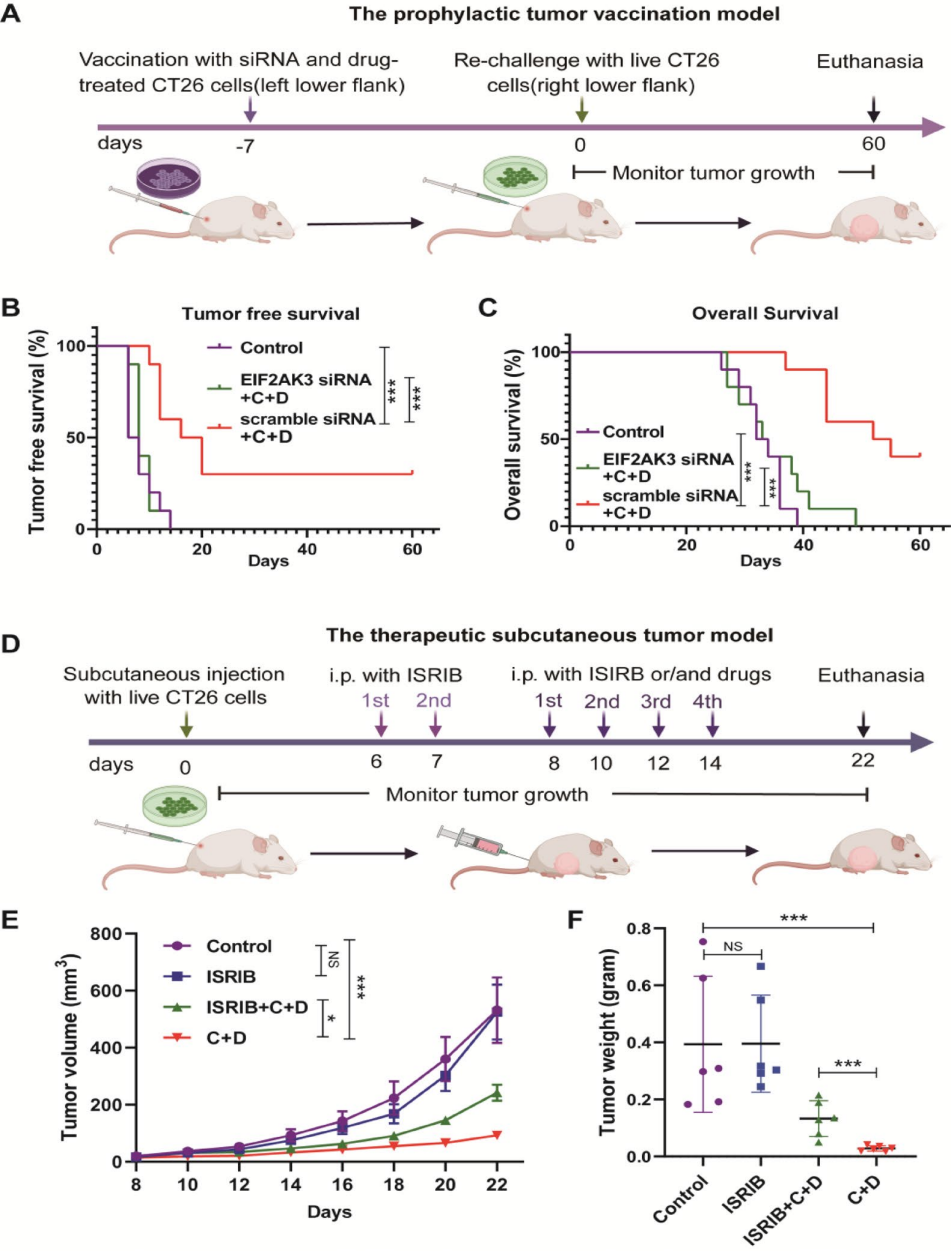
C + D treatment suppresses the tumor in a PERK/eIF2α-dependent manner. (**A**) Experimental design for the prophylactic CT26 tumor vaccination model with EIF2AK3 knockdown. CT26 cells were transfected with EIF2AK3 siRNA or scramble siRNA for 48 h, further treated with C + D for 20 h, and cultured with a fresh complete culture medium for 4 h. The above tumor cells were vaccinated into the BALB/c mice, which were subcutaneously rechallenged with live CT26 cells after 7 days. PBS without tumor cells was applied as the vaccine-negative control. The tumor initiation and progression were monitored for 60 days. (**B, C**) Tumor-free survival and overall survival of BALB/c mice after being inoculated with EIF2AK3 siRNA + C + D and scramble siRNA + C + D-treated CT26 tumor vaccine (*n* = 10). (**D**) Experimental design for the therapeutic subcutaneous CT26 tumor model with ISRIB combination. CT26 cells were subcutaneously injected to establish the tumor model, vector or 2.5 mg/Kg ISRIB dissolved in corn oil was intraperitoneal (i.p.) injected once a day for 2 days early, and then with ISRIB alone or the chemotherapy (3 mg/kg CDDP and 40 mg/kg DHA) combination every other day from the 8th day for four doses. Tumor growth was monitored for 22 days. (**E**) Tumor growth curves of BALB/c mice were monitored every other day after the C + D intervention (*n* = 6). (**F**) Weight of excised tumors of BALB/c mice after subcutaneous tumors were obtained from euthanized mice on the 22nd day (*n* = 6). The data are shown as mean ± SEM. For tumor weight, statistical analyses were performed with one-way ANOVA followed by Holm–Sidak’s multiple comparisons test after the log10 transformation. For tumor growth curves, *P* values were calculated by two-way ANOVA followed by Holm–Sidak’s multiple comparisons test. For Kaplan–Meier, *P* values were determined by the log-rank test. *P value < 0.05, ***P value < 0.001, NS *P* value > 0.05

Since C + D treatment activated the eIF2α phosphorylation and induced CALR exposure, we wondered whether C + D treatment suppresses tumor initiation and progression in a PERK/eIF2α-dependent fashion. Therefore, we knock down the expression of PERK in CT26 cells using EIF2AK3 siRNA (Figure S9). The EIF2ΑK3-knockdown CT26 cells were treated with C + D and then vaccinated into BALB/c mice as tumor vaccine. We observe the tumor initiation and growth after live CT26 cancer cells re-challenge (Fig. 7A). We found that knockdown of EIF2ΑK3 abolished the protective anti-tumor effect of the C + D-treated tumor vaccine compared to scramble siRNA (Fig. 7B, C, and Figure S10). Furthermore, we injected ISRIB into mice to inhibit the C + D-induced eIF2a phosphorylation and evaluated the anti-tumor effect of C + D treatment on established CT26 subcutaneous tumors (Fig. 7D). As shown in the growth curve and tumor weight at the endpoint, we found that ISRIB partially attenuated the tumor inhibition efficiency of C + D in vivo (Fig. 7E, F, and Figure S11). Overall, these findings suggested that PERK-mediated eIF2a phosphorylation is responsible for the enhanced anti-tumor effect of C + D treatment.

## Discussion

**Fig. 8:**
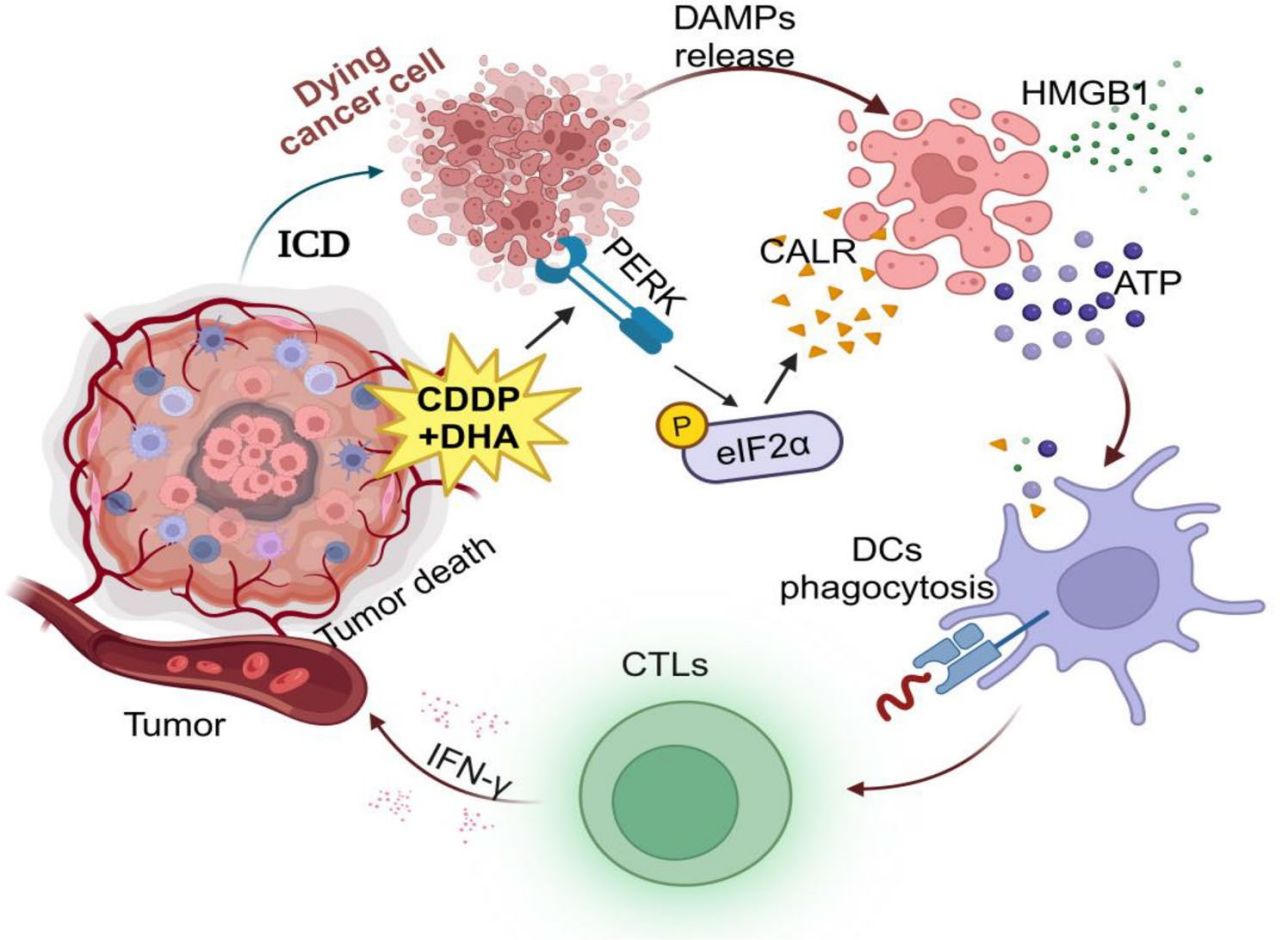
Schematic diagram of how DHA enhances anticancer immunosurveillance. DHA and cisplatin combination treatment ensures an optimal release of damage-associated molecular patterns (DAMPs) via the PERK/eIF2α pathway by dying cancer cells, which acts as immunostimulatory signals, promoting the activation of antigen-presenting cells (such as DCs) and cytotoxic T lymphocytes (CTLs), boosting their capacity to stimulate the immunosurveillance for cancer elimination

In both preclinical models and clinical settings, the induction of ICD has demonstrated significant efficacy in enhancing the long-term control of cancer through anti-tumor chemotherapy [5, 6, 10, 11]. While only a limited number of individual anticancer therapies possess the capacity to induce ICD, combining pharmacological interventions can restore the induction of ICD by triggering the release of various DAMPs [36]. Notably, the failure of CDDP to induce ICD can be reversed by combining it with agents such as cozotinib, pyridoxine, and cardiac glycosides [5, 18, 37]. The induction of ER stress is a prerequisite for ICD inducers [38]. The anti-tumor mechanisms of artemisinin derivatives and DHA include the induction of ROS and ER stress [28–30]. Here, our study elucidates that the combination therapy of cisplatin and DHA ensures optimal release of immunostimulatory signals (such as DAMPs) from dying cancer cells ensuring their capacity to stimulate immunosurveillance via the PERK/eIF2α pathway (Fig. 8). Furthermore, this combination therapy promotes the activation of antigen-presenting cells and cytotoxic T lymphocytes (CTLs), which is crucial for tumor recognition and elimination in immunosurveillance. The CDDP-induced ATP secretion level was elevated in CT26 cells, while failed in LLC cells, which may be attributed to the complex pathway involved in ATP secretion. Molecular mechanisms of ATP secretion involving distinct processes are as follows: (i) exocytosis of ATP-rich vesicles, (ii) liberation of cytosolic ATP molecules at gap junctions, (iii)gradient-driven efflux via oligomeric pannexin 1 (PANX1) channel [39]. The ICD-associated secretion of ATP relies on distinct processes such as apoptotic caspases (i.e., caspase 3 and caspase 7), autophagy (i.e., ATG5, ATG7 and BCN1), LAMP1-dependent lysosomal exocytosis (i.e., SNAP receptor, such as VAMP1 and VAMP7), membrane blebbing (i.e., ROCK1, myosin II) and plasma membrane permeabilization (i.e., PANX1) [40].

Our findings demonstrated that C + D treatment promoted the generation of ROS, cancer cell death, and the release of DAMPs, indicating that cancer cell death is immunogenic. Several studies demonstrates that DHA alone or combined with gefitinib/chlorin e6 against lung cancer by inducing ROS-dependent apoptosis and ferroptosis [41–44]. Moreover, DHA remodels macrophage into an M1 phenotype via ferroptosis, and DHA-loaded nanoreactor motivates anti-cancer immunotherapy by induced ferroptosis for reprogramming of macrophage [45, 46]. DHA exhibits synergistic therapeutic efficacy with cisplatin to induce ferroptosis in pancreatic ductal adenocarcinoma [47]. However, Chen et al. demonstrated that DHA induced unfolded protein response feedback attenuates ferroptosis via PERK/ATF4/HSPA5 pathway in glioma cells [48]. DHA also induces pyroptosis in breast and esophageal cancer cells [49, 50]. The cellular response of artemisinin and its derivatives towards cancer cells various cell death modes, such as apoptosis, autophagy, ferroptosis, necrosis, necroptosis, and oncosis, this may be attributed to cell specificity [51]. Moreover, Han and colleagues also found that DHA elicits immunogenic death through ferroptosis-triggered ER stress and DNA damage for lung cancer immunotherapy [52]. However, our data showed that cisplatin and DHA combination therapy function more efficiently than DHA alone, at least at the doses used in this study.

The gold standard for assessing ICD inducers is in vivo vaccination experiments, where tumor cells treated with chemotherapy drugs activate specific anti-tumor immune responses after being inoculated into mice of the same genotype. This process protects against the same type of live tumor cells, acting as a long-term vaccine [4, 53]. Our study demonstrated that the C + D-treated vaccine could extend tumor-free survival and provide specific protective anti-tumor immunity in immunocompetent mice. In contrast, the protective anti-tumor immunity of the C + D vaccine was absent in immunodeficient BALB/c-nu mice. This observation aligns with the concept of immunosurveillance, supported by previous findings showing that cancer develops more frequently in immune-deficient mice but loses its antigenic potential in immunocompetent hosts [12]. Our data, utilizing both immune-competent and immune-deficient mice, revealed that C + D treatment substantiates immune surveillance of cancer cells. In addition, using a therapeutic mouse model, we demonstrated that rapid tumor growth in immunodeficient mice, when compared to immunocompetent mice, highlights the distinctive immunosurveillance significance of DHA in cisplatin and DHA combination anticancer treatment.

Our study suggests that C + D therapy promotes the infiltration of CD4^+^ and CD8^+^ CTLs and elevates the ratio of CD8^+^ T cells to FOXP3^+^ Tregs, eliciting local anti-cancer immunity in TME and resulting in prolonged survival outcomes. Moreover, M1 macrophages in DLN were upregulated, and DCs in the spleen were activated after C + D treatment. In accordance with this observation, the accumulating evidence of the promoted immunosurveillance indicates that (i) cancers infiltrated with more positive immune effectors (such as CD8^+^ and CD4^+^ CTLs, NK cells, and activated DCs) and relatively fewer immunosuppressive cells (such as FOXP3^+^ Tregs and myeloid-derived suppressor cells) exhibit a favorable prognosis [12, 54, 55]; (ii) anti-cancer therapy (such as ICD inducers) is particularly efficient if local anti-cancer immunity is activated [12, 56]. DAMPs release promotes antigen-presenting cells (macrophages and DCs) to present tumor antigens to activated CD8^+^ T cells [57–59].

Some studies have found that the ER stress involved in ICD is PERK-dependent eIF2α phosphorylation related to CALR exposure [60, 61], and ER stress response-elicited CALR exposure may contribute to cancer immunosurveillance [62]. In concordance, our study identified that DHA promotes eIF2α phosphorylation, subsequently triggering CALR exposure, and consequently restores the immunogenicity of CDDP-induced cell death. CDDP fails to transfer CALR to the cell surface because of the deficiency of PERK-dependent eIF2α phosphorylation in ER stress [17]. Additionally, inhibiting eIF2α phosphorylation by pharmaceutical or genetic methods abolished C + D-induced CALR exposure in vitro and attenuated the anti-tumor efficiency of C + D treatment in vivo. Our study also highlights the importance of the PERK-mediated eIF2a phosphorylation-induced CALR exposure for anticancer immunosurveillance and enhanced anti-tumor effect of C + D treatment.

Clinical trials have shown that the anti-tumor effects of artemisinin and its derivatives on cervical cancer, advanced NSCLC, metastatic breast cancer, and colorectal cancer are being translated into clinical practice [63–65]. A phase II clinical trial explored the tumor inhibition effect of the combination treatment of DHA with icotinib in EGFR-positive NSCLC patients (NCT03402464) [30]. Therefore, CDDP plus DHA therapy holds promise as an anti-tumor strategy for patients with cancer in the future with excellent safety and efficiency profiles.

However, further investigation of ER stress in immune activation in vivo will better demonstrate the mechanistic details of C + D treatment. Before applying this strategy to clinical practice, more research and clinical trials are needed to determine its direct efficacy in human individuals. It will also be essential to evaluate the anti-cancer effects of C + D therapy on various types of cancer in future clinical trials.

## Conclusion

In conclusion, our findings revealed that DHA enhances the anti-tumor efficacy of cisplatin treatment, which is attributed to driven ICD and anti-cancer immunosurveillance. Mechanistically, DHA restored the immunogenicity of CDDP-induced dying tumor cells by activating the PERK/eIF2α pathway to induce CALR exposure. The combination therapy of cisplatin and DHA effectively restored CTLs response in TME, leading to heightened immunosurveillance and improved tumor control in a PERK/eIF2α-dependent manner. This study presents a novel and potential strategy for improving anti-tumor efficacy in clinical practice through the combination of cisplatin with dihydroartemisinin.

### Electronic supplementary material

Below is the link to the electronic supplementary material.

Supplementary Material 1

## Data Availability

All data generated or analyzed during this study are included in this published article or uploaded as supplementary information.

## Abbreviations

7-AAD: 7-amino actinomycin D
ATP: Adenosine Triphosphate
BMDCs: Bone Marrow-Derived Dendritic Cells
CDDP: Cis-Dichlorodiammineplatinum; Cisplatin
C + D: Combination of Cisplatin and Dihydroartemisinin
CLSM: Confocal Laser Scanning Microscopy
CALR: Calreticulin
CT26: A Murine Colorectal Carcinoma
CTLs: Cytotoxic T Lymphocytes
DAMPs: Damage-Associated Molecular Patterns
DCs: Dendritic Cells
DHA: Dihydroartemisinin
DiL: 1,1’-dioctadecyl-3,3,3’,3’-tetramethylindocarbocyanine perchlorate;
DiO: 3,3’-Dioctadecyloxacarbocyanine Perchlorate
DLN: Draining Lymph Node
DMF: N, N-Dimethylformamide
DMSO: Dimethyl Sulfoxide
eIF2α: Eukaryotic Translation Initiation Factor 2α
ELISA: Enzyme-Linked Immunosorbent Assay
ER: Endoplasmic Reticulum
FBS: Fetal Bovine Serum
H&E: Hematoxylin and Eosin
HMGB1: High Mobility Group Protein B1
ICD: Immunogenic Cell Death
IFN-γ: Interferon-Gamma
IHC: Immunohistochemical
i.p.: Intraperitoneal
ISRIB: Integrated Stress Response Inhibitor
LLC: Lewis Lung Carcinoma
PANX1: Pannexin 1
PERK: Protein kinase RNA–like Endoplasmic Reticulum Kinase
ROS: Reactive Oxygen Species
Tha: Thapsigargin
TILs: Tumor-Infiltrating Lymphocytes
TME: Tumor Microenvironment
Tregs: Regulatory T cells
TUNEL: Terminal Deoxynucleotidyl Transferase-Mediated dUTP Nick-End Labeling
NSCLC: Non-Small Cell Lung Cancer
