# Supplementary material for: Dihydroartemisinin restores the immunogenicity and enhances the anticancer immunosurveillance of cisplatin by activating the PERK/eIF2α pathway
Source: Cell Biosci. 2024 Aug 1;14:100. doi: 10.1186/s13578-024-01254-0 (PMC11295430; doi:10.1186/s13578-024-01254-0)
Supplement: Supplementary file 1 — Supplementary Material 1 [file 13578_2024_1254_MOESM1_ESM.docx]

Supplementary information

**Dihydroartemisinin restores the immunogenicity and enhances the anticancer immunosurveillance of cisplatin by activating the PERK/eIF2α pathway**

Yumei Li^1, 2,3 #^, Pei Ma^1, 4, #^, Jingxia Li^1, 2,3^, Feng Wu^1, 2,3^, Mengfei Guo^1, 2,3^, E Zhou^1, 2,3^, Siwei Song^1, 2,3^, Sufei Wang^1, 2,3^, Shuai Zhang^1, 2,3 *^, Yang Jin^1, 2, 3*^

1. Department of Respiratory and Critical Care Medicine, Hubei Province Clinical Research Center for Major Respiratory Diseases, Key Laboratory of Pulmonary Diseases of National Health Commission, Union Hospital, Tongji Medical College, Huazhong University of Science and Technology, Wuhan, Hubei, 430022, China.

2. The Ministry of Education Key Laboratory of Biological Targeted Therapy, Union Hospital, Tongji Medical College, Huazhong University of Science and Technology

3. Hubei Province Engineering Research Center for Tumor-Targeted Biochemotherapy, Union Hospital, Tongji Medical College, Huazhong University of Science and Technology

4. State Key Laboratory of Respiratory Disease, The First Affiliated Hospital of Guangzhou Medical University, Guangzhou, China.

^#^ Yumei Li and Pei Ma contributed equally to this work.

^*^ Correspondence should be addressed to Shuai Zhang 2018xh0137@hust.edu.cn and Yang Jin [whuhjy@126.com](mailto:whuhjy@126.com).

**Table S1.** Antibodies used for flow cytometry of tumor-infiltrating lymphocytes

**Figure S1.** Phagocytosis of drug-treated tumor cells by BMDCs.

**Figure S2.** Excised tumors and the body weight change of BALB/c mice.

**Figure S3.** Tumor growth and prognosis of LLC tumor-bearing C57 mice after different treatments.

**Figure S4.** Excised tumors from BALB/c-nu mice after euthanized on the 18th day.

**Figure S5.** Gating strategy of flow cytometry analysis for detecting tumor-infiltrating T lymphocytes of the CT26 tumor-bearing BALB/c mice after different treatments.

**Figure S6.** Flow cytometry analysis for detecting macrophage from the draining lymph nodes (DLN) of the CT26 tumor-bearing BALB/c mice.

**Figure S7.** Flow cytometry analysis for detecting dendritic cells (DCs) from the spleen of the CT26 tumor-bearing BALB/c mice.

**Figure S8.** Full and uncropped western blots for Figure 6.

**Figure S9.** Relative expression of PERK was determined by Western blot after EIF2ΑK3 knockdown of CT26 cells.

**Figure S10.** Tumor growth curves of CT26 tumor in prophylactic vaccination model with EIF2AK3 knockdown.

**Figure S11.** Excised tumors from the euthanized BALB/c mice on the 22nd day after the ISRIB combination.

**Supplementary tables**

**Table S1**. Antibodies used for flow cytometry of tumor-infiltrating lymphocytes

| Antibodies | Clone | Source | Identifier |
| --- | --- | --- | --- |
| APC/Cyanine7 anti-mouse CD45 | 30-F11 | BioLegend | 103116 |
| PE/ Cyanine7 anti-mouse I-A/I-E | M5/114.15.2 | BioLegend | 107630 |
| APC anti-mouse IFN-γ | XMG1.2 | BioLegend | 505810 |
| Brilliant Violet 605™ anti-mouse/human CD11b | M1/70 | BioLegend | 101237 |
| FITC anti-mouse CD11c | N418 | BioLegend | 117306 |
| APC anti-mouse CD206 (MMR) | C068C2 | BioLegend | 141708 |
| PE-CF594 Rat Anti-Mouse CD8a | 53-6.7 | BD Pharmingen | 562283 |
| PE Rat Anti-Mouse F4/80 | T45-2342 | BD Pharmingen | 565410 |
| BV421 Rat Anti-Mouse CD86 | GL1 | BD Pharmingen | 564198 |
| PE anti-mouse FOXP3 | NRRF-30 | eBioscience | 12-4771-82 |
| Super Bright 600 anti-mouse CD4 | RM4-5 | eBioscience | 63-0042-82 |
| PerCP-Cyanine5.5 anti-mouse CD25 | PC61.5 | eBioscience | 45-0251-82 |
| TruStain FcX (anti-mouse CD16/32) | 93 | BioLegend | 101319 |
| Zombie Aqua Fixable Viability Kit | NA | BioLegend | 423101 |

**Supplementary figures**


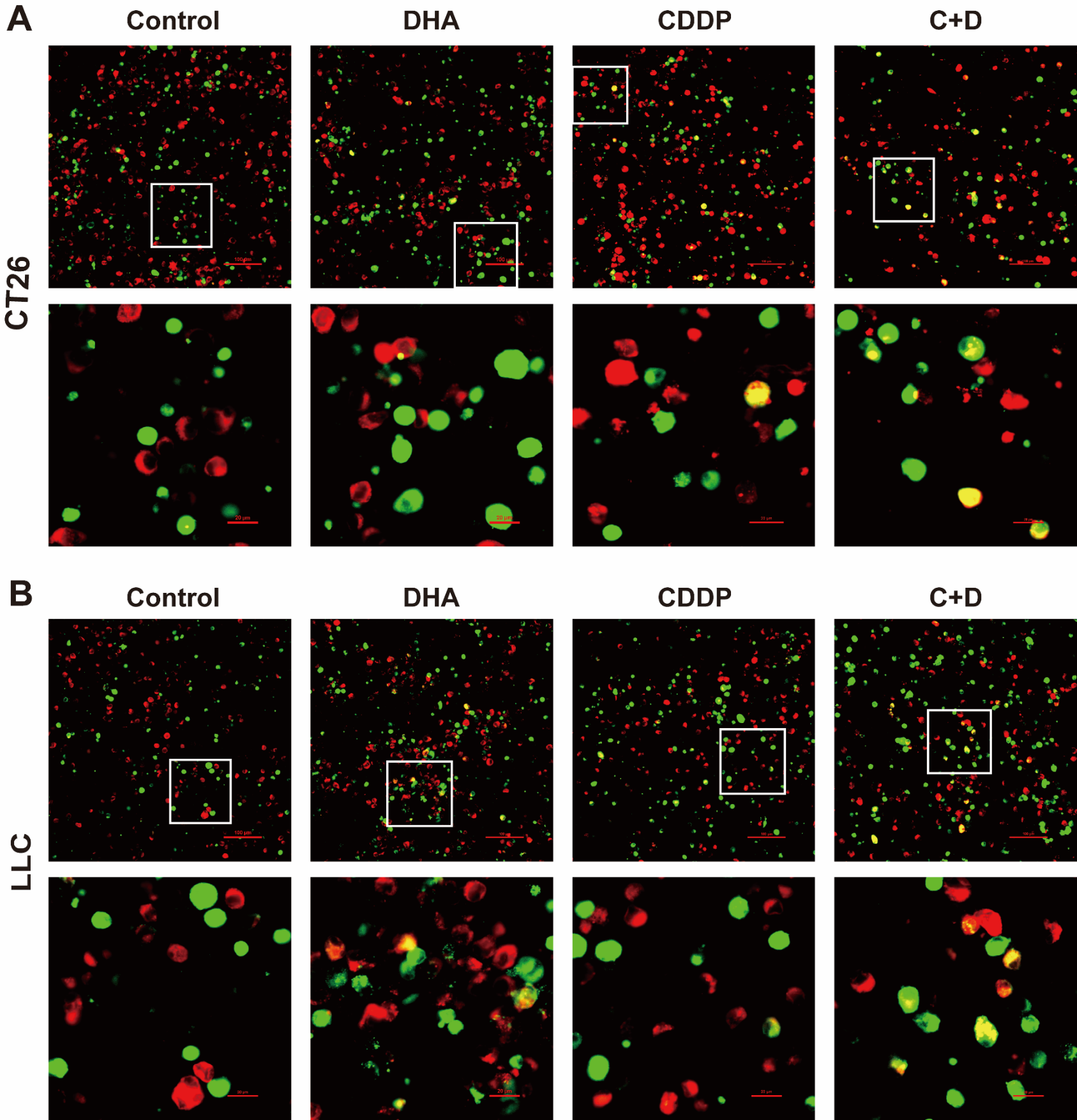


**Figure S1.** Phagocytosis of drug-treated tumor cells by BMDCs. The uptake of drug-treated (**A**) CT26 cells and (**B**) LLC cells was identified by representative images under CLSM (the upper panels scale bar, 100 μm; the lower panels scale bar, 20 μm).


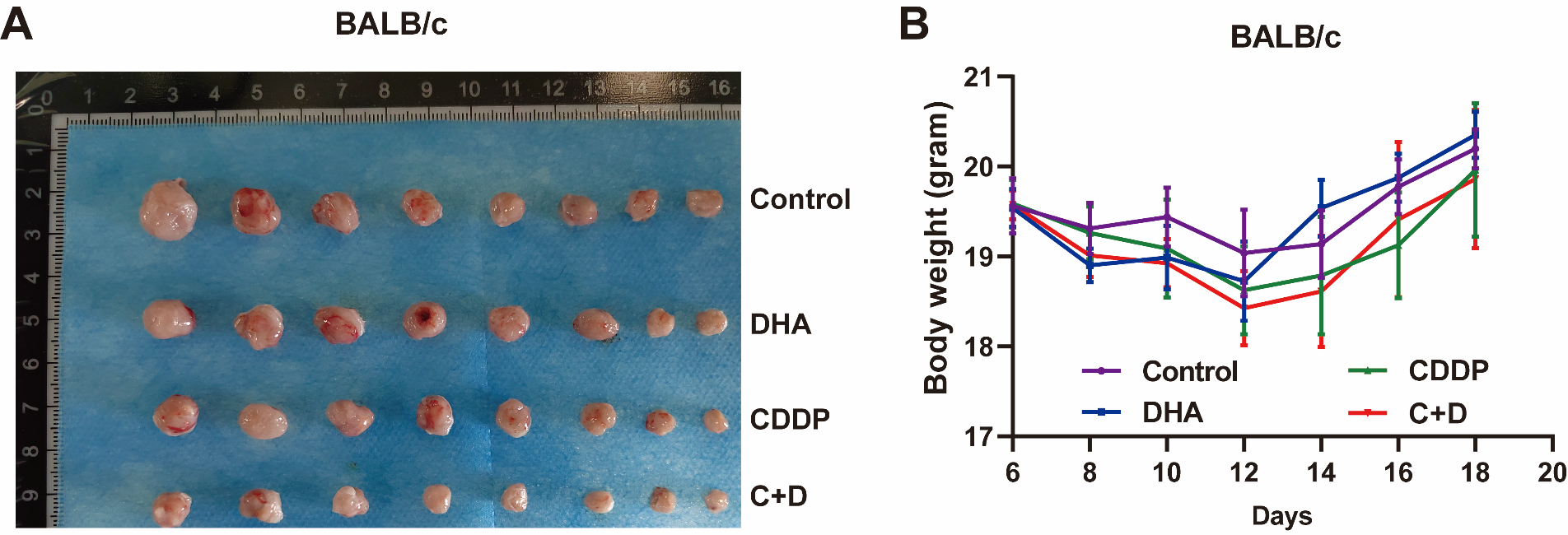


**Figure S2.** Excised tumors and the body weight change of BALB/c mice. (**A**) Image of excised tumors from BALB/c mice after euthanized on the18th day (*n* = 8). (**B**) Body weight change of mice in BALB/c mice during the different treatments.


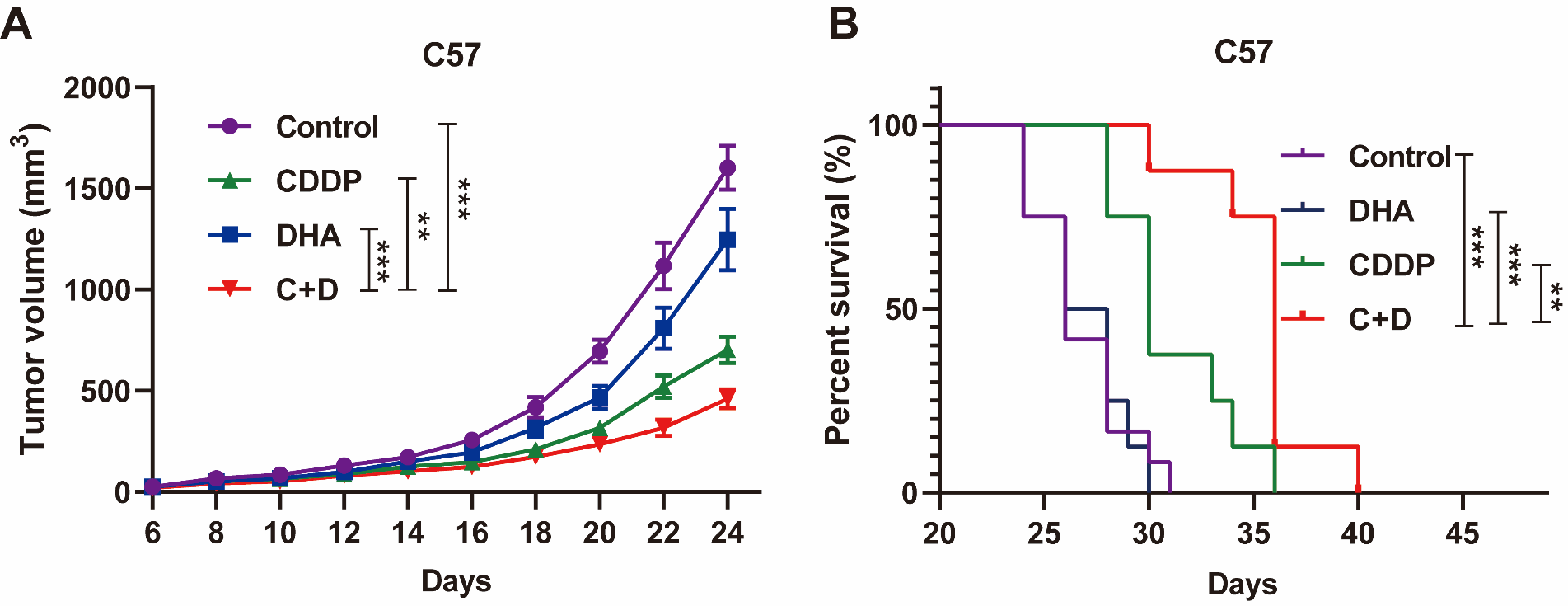


**Figure S3.** Tumor growth and prognosis of LLC tumor-bearing C57 mice after different treatments. (**A**) The tumor growth curves. (**B**) Kaplan–Meier survival curves. Data are presented as mean ± SEM (*n* = 8). For tumor growth curves, *P* values were calculated by two-way ANOVA followed by Holm–Sidak’s multiple comparisons test. For Kaplan–Meier, *P* values were determined by the log-rank test. * *P* value < 0.05, ** *P* value <0.01, *** *P* value < 0.001


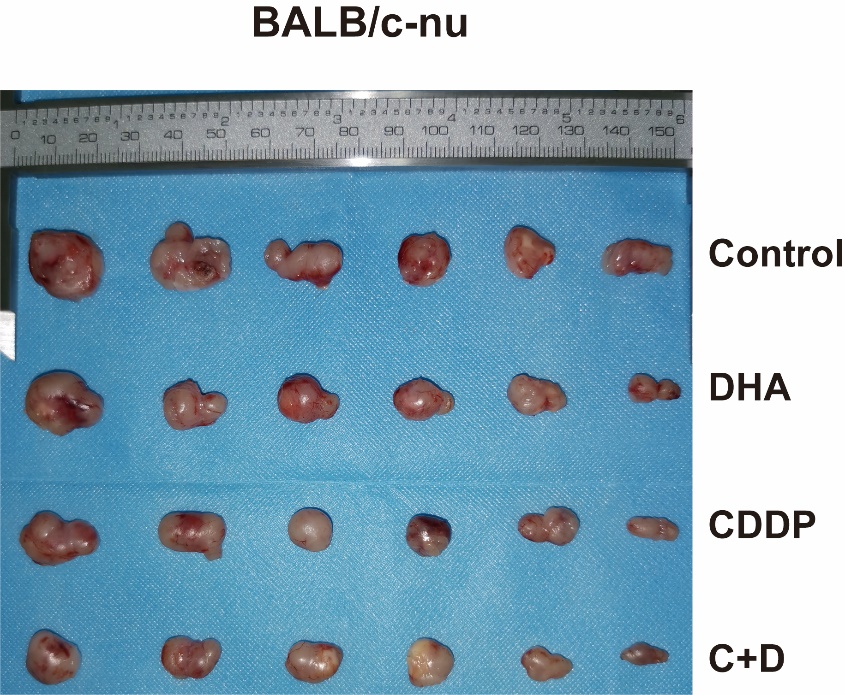


**Figure S4.** Excised tumors from BALB/c-nu mice after euthanized on the 18th day (*n* = 6).


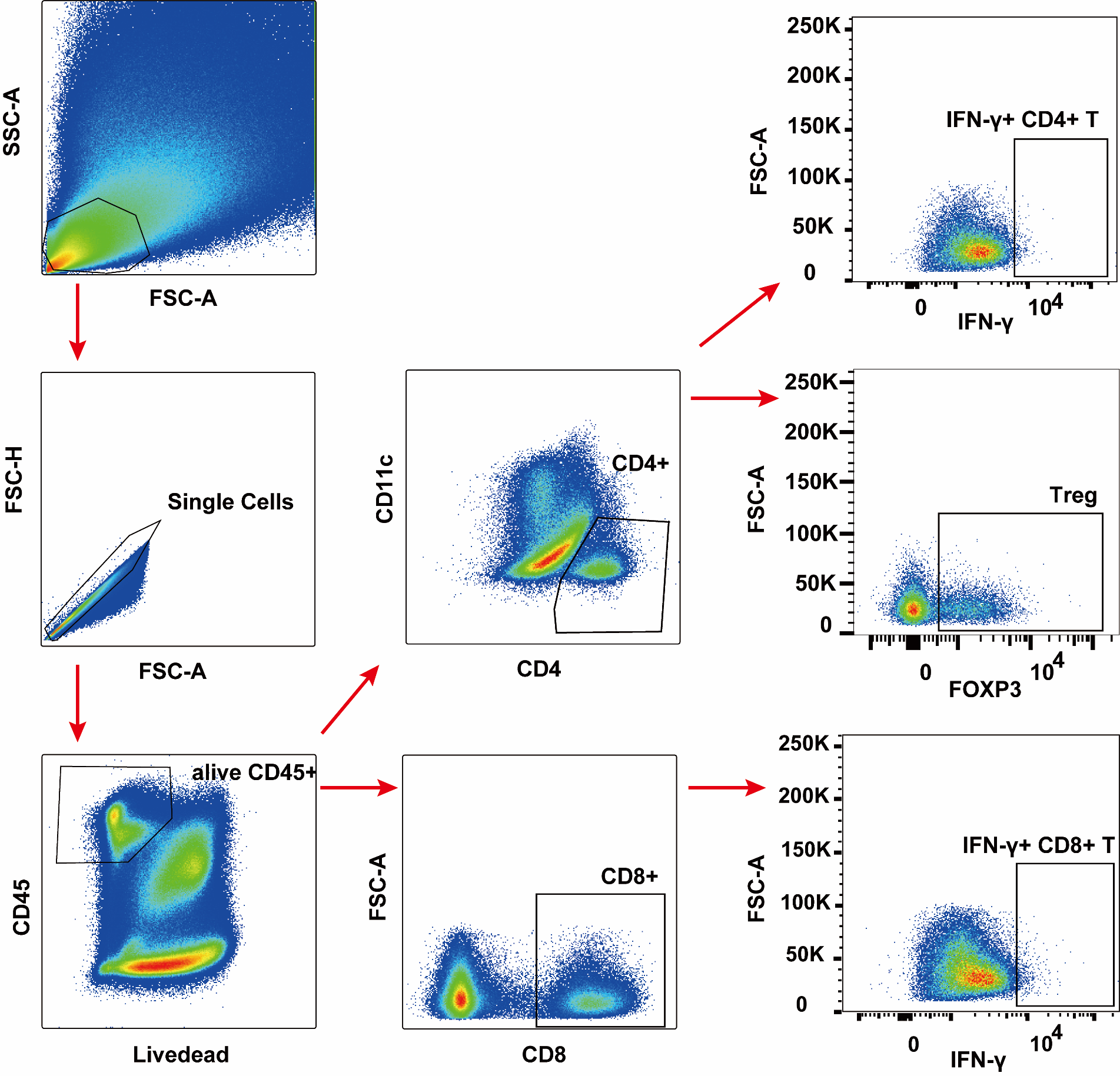


**Figure S5.** Gating strategy of flow cytometry analysis for detecting tumor infiltrating T lymphocytes of the CT26 tumor-bearing BALB/c mice after different treatments. The negative control of the IFN-γ gating strategy was supplied above.


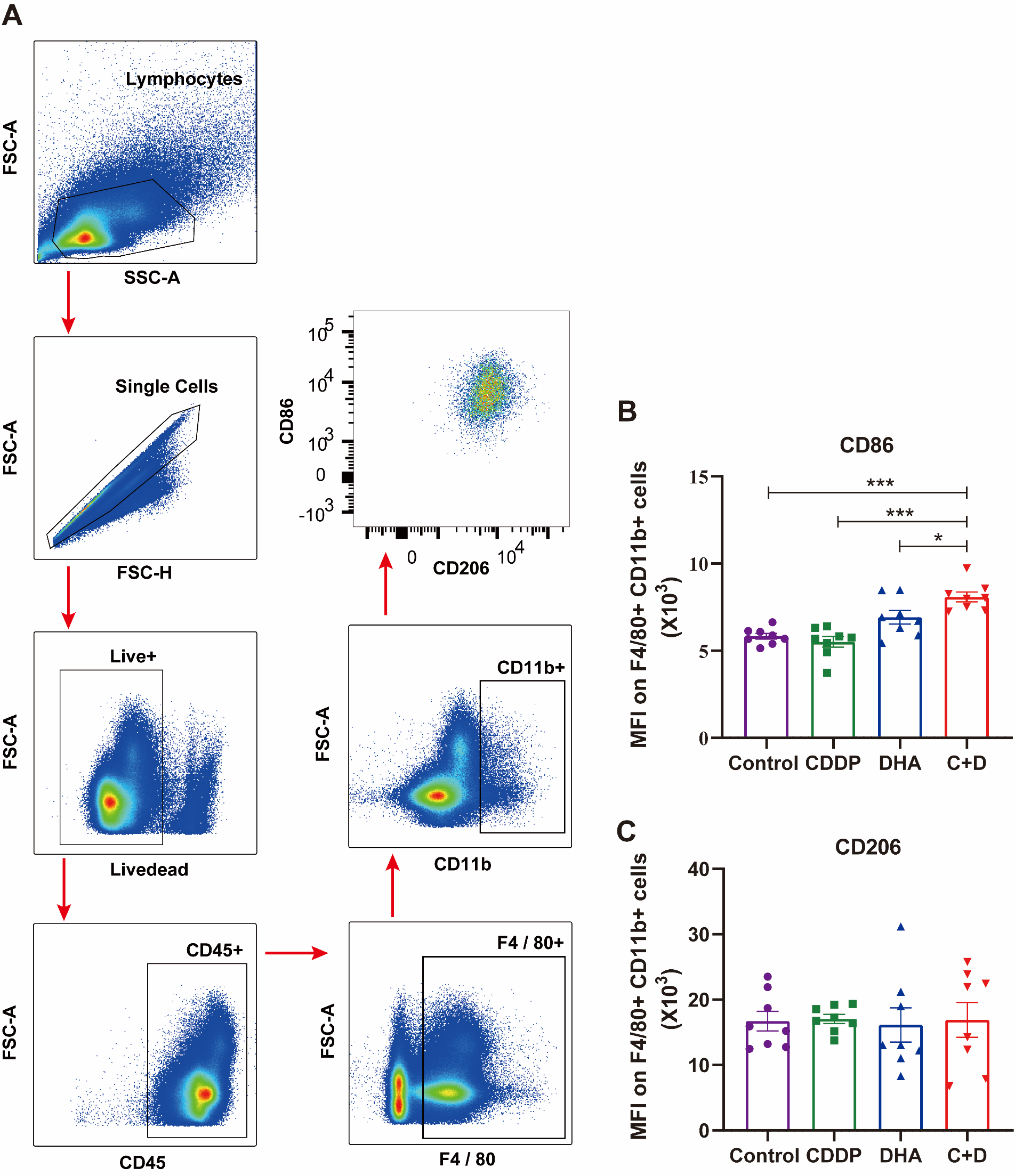


**Figure S6.** Flow cytometry analysis for detecting macrophage from the draining lymph nodes (DLN) of the CT26 tumor-bearing BALB/c mice. (**A**) Gating strategy of flow cytometry analysis. Flow cytometry analysis of the changes of (**B**) CD86 level and (**C**) CD206 level in the F4/80^+^CD11b^+^ macrophages in DLN. Data are presented as mean ± SEM (*n* = 8), and statistical analyses were performed with one-way ANOVA followed by the Holm–Sidak’s multiple comparisons test. **P* value < 0.05, ****P* value < 0.001


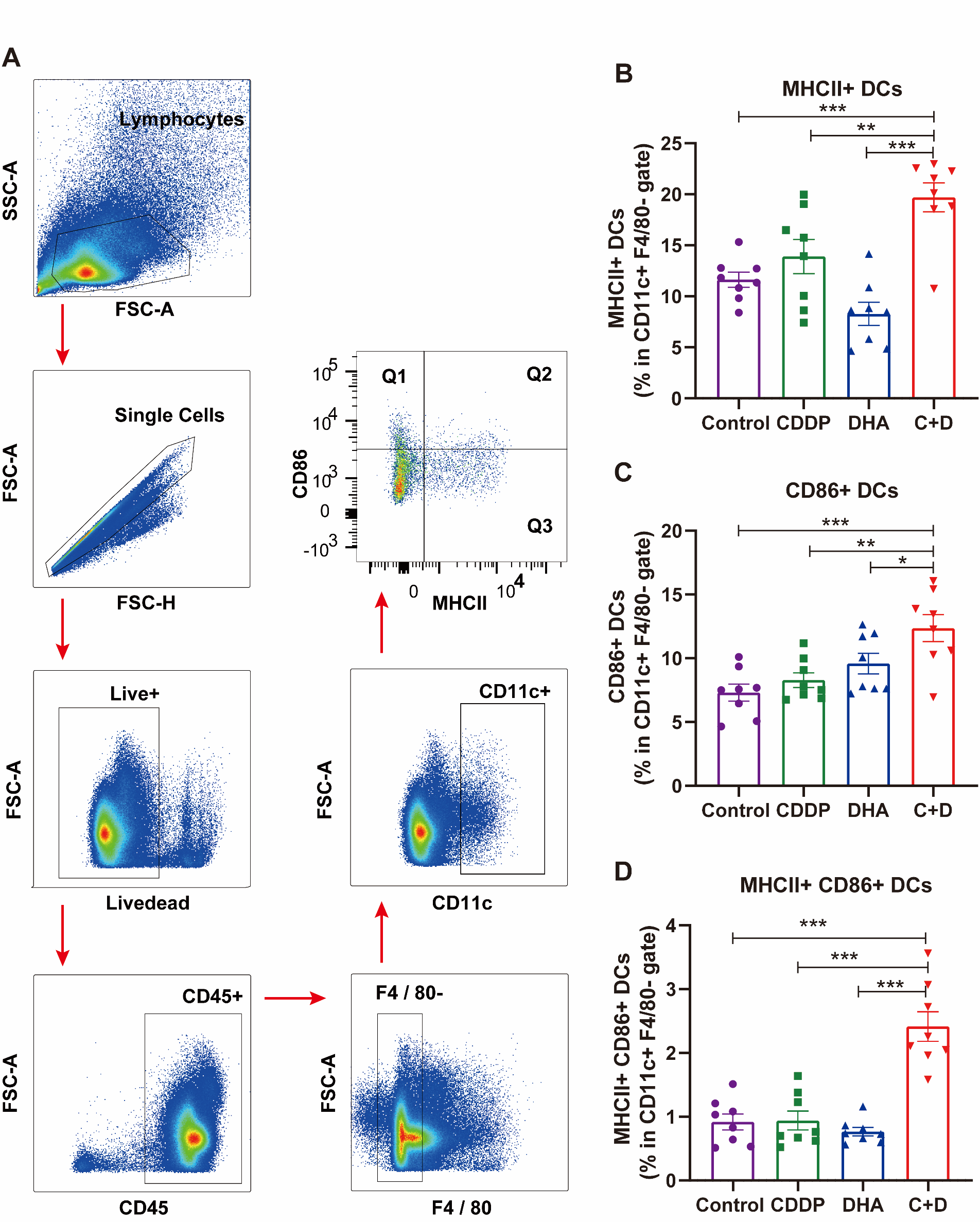


**Figure S7.** Flow cytometry analysis for detecting dendritic cells (DCs) from the spleen of the CT26 tumor-bearing BALB/c mice. (**A**) Gating strategy of flow cytometry analysis. Statistic results for the proportions of (**B**) MHCII^+^ DCs, (**C**) CD86^+^ DCs, and (**D**) MHCII^+^CD86^+^ DCs in CD11c^+^F4/80^-^ cells in the spleen. The data are shown as mean ± SEM (*n* = 8), and statistical analyses were performed with one-way ANOVA followed by the Holm–Sidak’s multiple comparisons test. **P* value < 0.05, ***P* value <0.01, ****P* value < 0.001


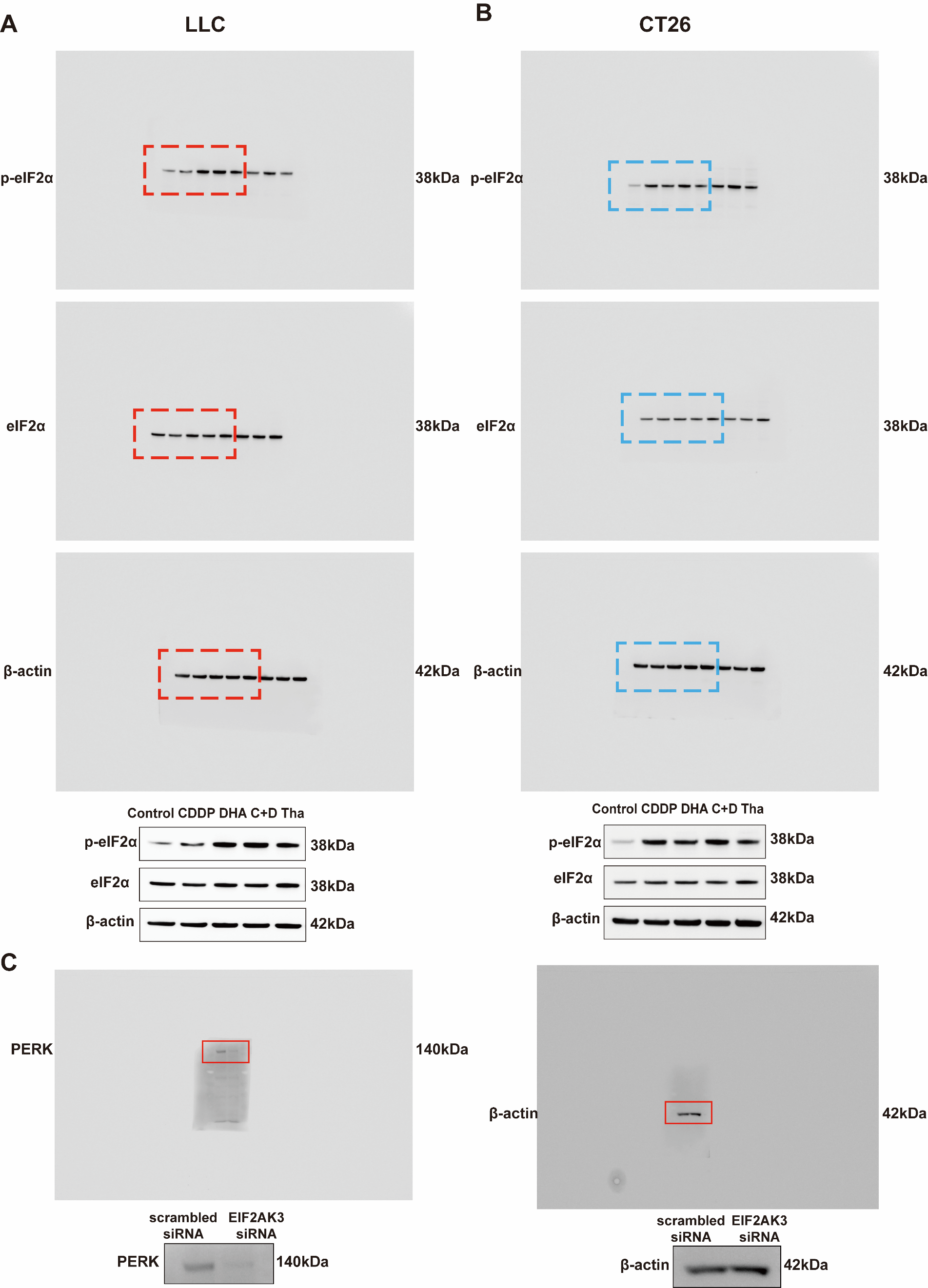


**Figure S8.** Full and uncropped western blots for Figure 6. (**A–C**) Full and uncropped western blots for Figure 6A (red), Figure 6C (blue), and Figure 6G.


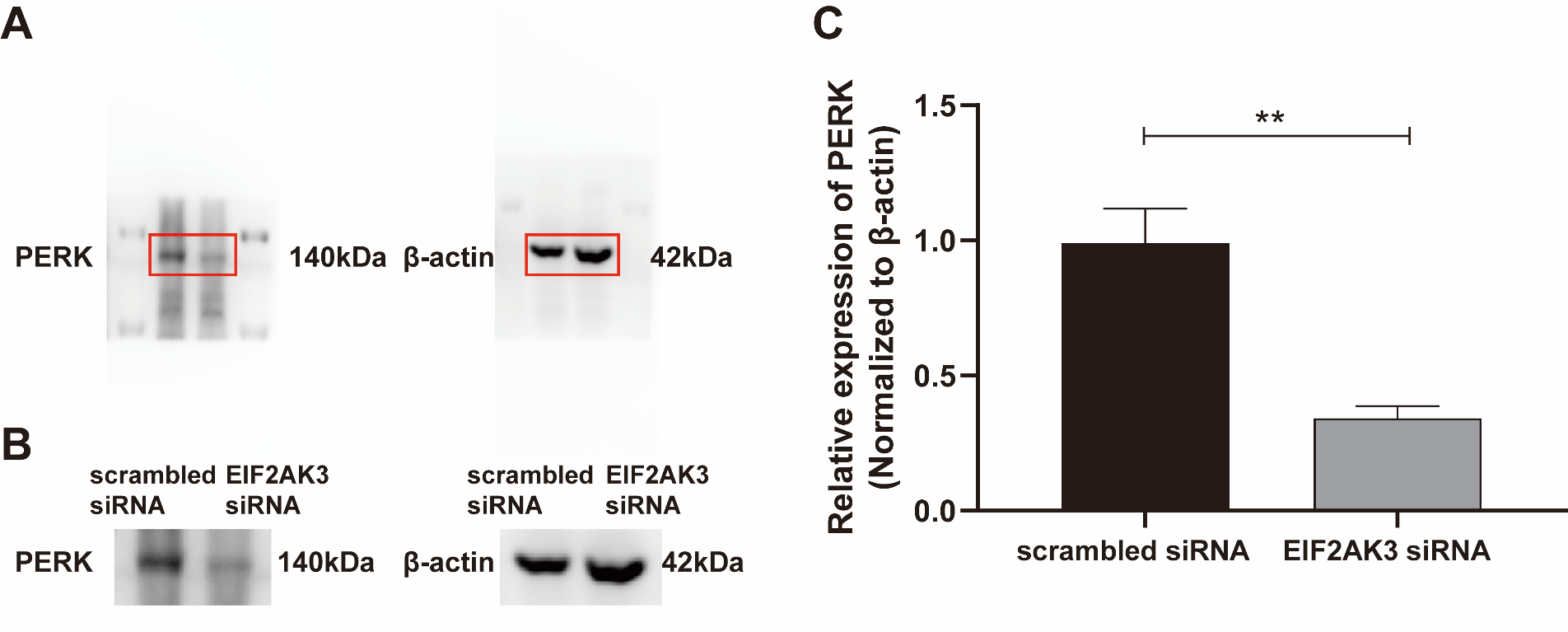


**Figure S9.** The relative expression of PERK was determined by Western blot after EIF2ΑK3 knockdown of CT26 cells. (**A**) Full and (**B**) uncropped western blots for PERK and β-actin. (**C**) The relative expression of PERK in CT26 cells after knockdown of EIF2AK3. These data are shown as mean ± SEM (*n* = 3), and statistical analyses were performed with an unpaired Student’s t-test. ***P* value <0.01.


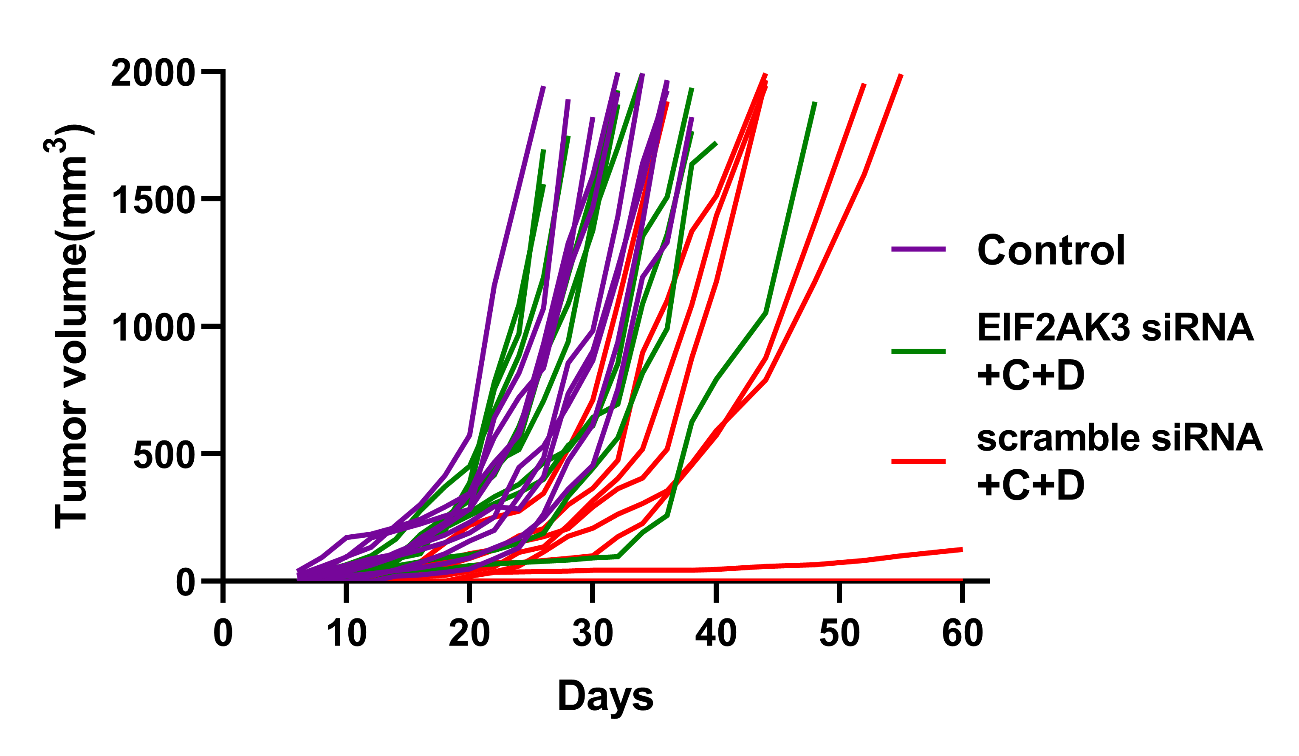


**Figure S10.** Tumor growth curves of CT26 tumors in prophylactic vaccination model with EIF2AK3 knockdown (*n* = 10). When inoculated with the scramble siRNA + C + D-treated CT26 tumor vaccine, three of ten BALB/c mice still did not form tumors during the observation period after live tumor cells rechallenge.


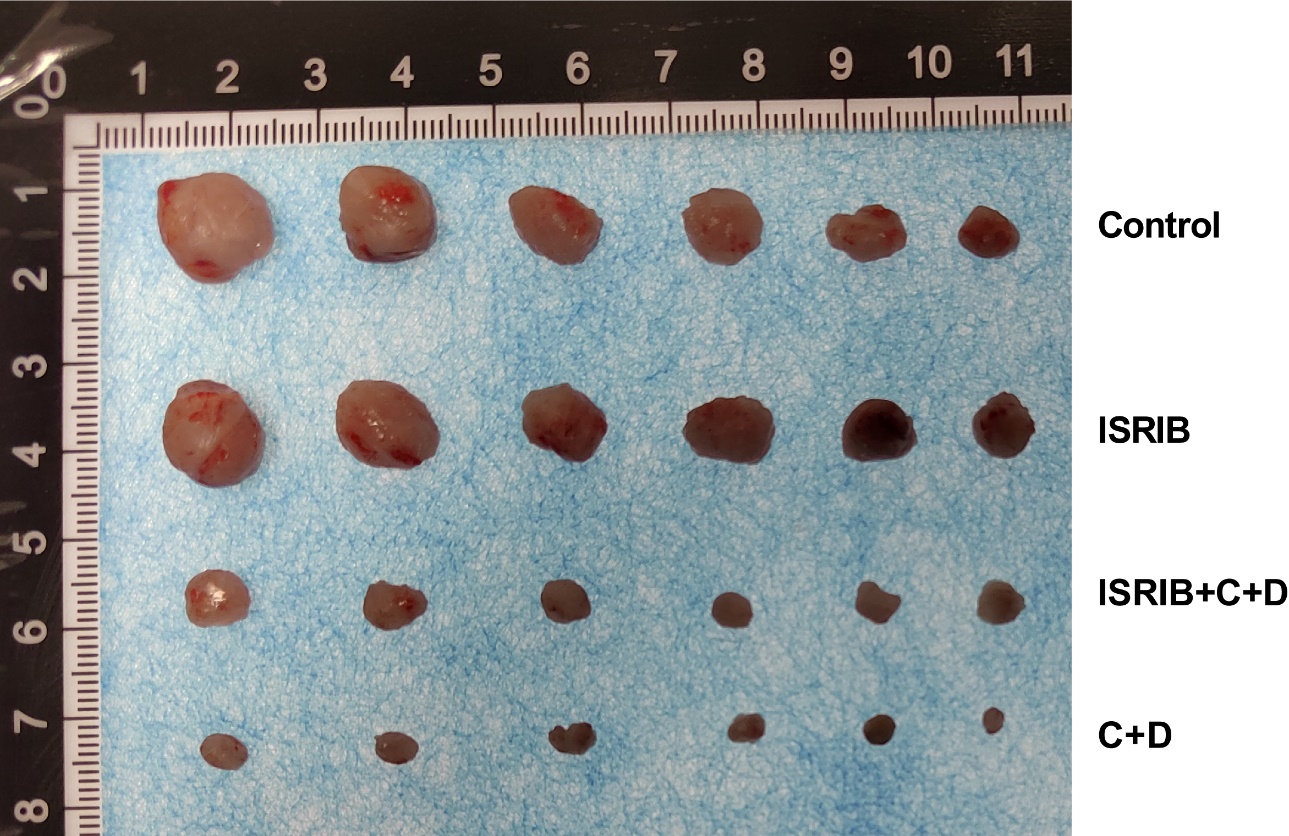


**Figure S11.** Excised tumors from the euthanized BALB/c mice on the 22nd day after the ISRIB combination (*n* = 6).
